# Injurious Fall Risk Differences Among Older Adults With First-Line Depression Treatments

**DOI:** 10.1001/jamanetworkopen.2024.35535

**Published:** 2024-08-26

**Authors:** Grace Hsin-Min Wang, Edward Chia-Cheng Lai, Amie J. Goodin, Rachel C. Reise, Ronald I. Shorr, Wei-Hsuan Lo-Ciganic

**Affiliations:** 1 Department of Pharmaceutical Outcomes and Policy, College of Pharmacy, University of Florida, Gainesville; 2 School of Pharmacy, Institute of Clinical Pharmacy and Pharmaceutical Sciences, College of Medicine, National Cheng Kung University, Tainan, Taiwan; 3 North Florida/South Georgia Veterans Health System Geriatric Research Education and Clinical Center, Gainesville, Florida; 4 College of Public Health and Health Professions and College of Medicine, University of Florida, Gainesville; 5 Division of General Internal Medicine, School of Medicine, University of Pittsburgh, Pennsylvania; 6 Center for Pharmaceutical Policy and Prescribing, Health Policy Institute, University of Pittsburgh, Pennsylvania

## Abstract

**Importance:**

One-third of older adults in the US have depression, often treated with psychotherapy and antidepressants. Previous studies suggesting an increased risk of falls and related injuries (FRI) associated with antidepressant use may be affected by confounding by indication or immortal time bias.

**Objective:**

To evaluate the association between FRI risk and first-line treatments in older adults with depression.

**Design, Setting, and Participants:**

This cohort study used a target trial emulation framework with a cloning-censoring-weighting approach with Medicare claims data from 2016 to 2019. Participants included fee-for-service beneficiaries aged 65 years or older with newly diagnosed depression. Data were analyzed from October 1, 2023, to March 31, 2024.

**Exposures:**

First-line depression treatments including psychotherapy, sertraline, escitalopram, citalopram, mirtazapine, duloxetine, trazodone, fluoxetine, bupropion, paroxetine, and venlafaxine.

**Main Outcome and Measure:**

One-year FRI rate, restricted mean survival time (RMST), and adjusted hazard ratio (aHR) with 95% CI.

**Results:**

Among 101 953 eligible beneficiaries (mean [SD] age, 76 [8] years), 63 344 (62.1%) were female, 7404 (7.3%) were Black individuals, and 81 856 (80.3%) were White individuals. Compared with the untreated group, psychotherapy use was not associated with FRI risk (aHR, 0.94 [95% CI, 0.82-1.17]), while other first-line antidepressants were associated with a decreased FRI risk (aHR ranged from 0.74 [95% CI, 0.59-0.89] for bupropion to 0.83 [95% CI, 0.67-0.98] for escitalopram). The FRI incidence ranged from 63 (95% CI, 53-75) per 1000 person-year for those treated with bupropion to 87 (95% CI, 83-90) per 1000 person-year for those who were untreated. The RMST ranged from 349 (95% CI, 346-350) days for those who were untreated to 353 (95% CI, 350-356) days for those treated with bupropion.

**Conclusions and Relevance:**

In this cohort study of older Medicare beneficiaries with depression, first-line antidepressants were associated with a decreased FRI risk compared with untreated individuals. These findings provide valuable insights into their safety profiles, aiding clinicians in their consideration for treating depression in older adults.

## Key Points

**Question:**

How are first-line depression treatments associated with the risk of falls and related injuries (FRI) compared with no treatment in older adults with depression?

**Findings:**

This cohort study of 101 953 eligible beneficiaries found that first-line antidepressants were associated with a decreased FRI risk compared with no treatment in older adults with depression.

**Meaning:**

These findings suggest that first-line antidepressants were associated with a decreased FRI and may provide valuable insights into the safety profiles of these treatments, aiding clinicians in their consideration for treating depression.

## Teaser

This cohort study assesses the association between the risk of fall-related injuries and first-line treatments for depression among older US adults with depression.

## Introduction

In the US, depression is among the most common mental disorders in older adults, with a prevalence of approximately 32%.^1^ Depression-related symptoms, such as executive dysfunction, cognitive impairment, impaired sleep, nutritional deficiency, and gait status, can increase the risk of falls and related injuries (FRI), such as fractures and sprains.^2,3,4^ FRI commonly occur among older adults, imposing a substantial public health burden in the US. Each year, an average of 714 falls and 170 FRI are reported per 1000 adults aged 65 years or older, resulting in more than 32 000 deaths, 3 million emergency department (ED) visits, and 300 000 hospitalizations.^5,6^

The American College of Physicians recommends monotherapy with cognitive behavioral therapy or a second-generation antidepressant (AD) as the initial treatment for depression.^7^ While ADs are effective in treating depression, their adverse effects can affect alertness, balance, and blood pressure regulation, particularly in older adults, potentially increasing FRI risk.^8^ A meta-analysis reported that AD use was associated with an increased FRI risk compared with no use (odds ratio [OR], 1.57; 95% CI, 1.43-1.74) among older adults.^9^ In addition, the 2019 American Geriatric Society Beers Criteria recommended against using ADs in older adults with a history of falls or fractures.^10^ This recommendation may not be clinically actionable considering the benefit of ADs in managing depressive symptoms. Ideally, using ADs with a lower risk of FRI may provide safer treatment options for patients at risk of FRI. However, the Beers Criteria also stated that “There is no compelling evidence that certain AD confers less FRI risk than other ADs,” making depression treatment challenging for older adults.

Determining the optimal treatment for older adults with depression is complex because depression is also an independent risk factor for FRI.^2,3,4^ To our knowledge, no study has evaluated the association between psychotherapy and FRI risk. Observational studies comparing individuals who used ADs with those who did not use ADs are susceptible to confounding by depression (as the FRI event may be caused by the underlying depression for which the drug was prescribed instead of the drug itself) and immortal time bias (as patients may not have initiated ADs on the date of their first depression diagnosis).^11,12^ The target trial emulating (TTE) framework,^13^ followed by the cloning-censoring-weighting (CCW) approach has been proposed by Hernan et al^14^ to address these issues. To our knowledge, only 1 study has compared FRI risk among individual ADs (no significant difference in the risk of falls between sertraline and citalopram/escitalopram),^15^ while most studies compared between AD subgroups. Given the significance of understanding FRI risks associated with various first-line depression treatments and the current evidence gap, we aimed to evaluate the FRI risk among psychotherapy and first-line ADs compared with no treatment in older adults with depression.

## Methods

### Data Sources

We used a 15% modified sample of fee-for-service beneficiaries from Medicare Parts A, B, and D claims data from 2016 to 2019, with an oversampling of individuals from Florida. Medicare provides coverage for approximately 97% of the US population aged 65 years or older. This study adhered to the Strengthening the Reporting of Observational Studies in Epidemiology (STROBE) reporting guideline^16^ and was approved by the University of Florida institutional review board. Informed consent was waived due to the retrospective collection of anonymized data.

### Study Design and Cohort Selection

This was a target emulated trial with retrospective cohort study design. Our study design followed the TTE framework, which provides a structured process for avoiding common methodologic pitfalls in observational studies in order to yield the same estimates as the target pragmatic trials.^17^ The key components of TTE include eligibility criteria, treatment strategies, assignment procedures, follow-up, outcome, causal contrasts, and analysis plan (eTable 1 in Supplement 1).^17^

We included beneficiaries diagnosed with depression in 2017 or 2018, and the index date (day 0) was defined as the first diagnosis date. We excluded beneficiaries who: (1) were aged younger than 65 years; (2) were not US citizens; (3) used hospice services or were in a nursing facility within day −365 to day 0 (focusing on older adults who were not in institutions as nursing home residents are often weaker and may receive additional care); (4) did not have continuous enrollment within day −365 to day 0; (5) had depression within day −365 to day −1; (6) received psychotherapy or any AD prescription within day −365 to day −1; (7) experienced FRI events within day −365 to day 0; and (8) received more than 1 first-line depression treatment on day 0. Depression was defined by the presence of diagnosis codes at any position in any setting according to the Chronic Conditions Warehouse Algorithms.^18^ Beneficiaries were followed until the earliest of FRI occurrence, death, transition to Medicare Advantage plans, hospice services or nursing facility use, treatment switching, combination, or discontinuation, or the end of the 1-year follow-up (eFigure 1 in Supplement 1). Treatment discontinuation was defined as 14 days after the presumed end date of the last prescription. One year follow-up was chosen based on 2 systematic reviews assessing the impact of different medications on falls in elderly populations.^19,20^ Most of the included observational studies followed patients for 1 year, while others ranged from 6 months to 3 years.

### Exposure

Our exposure of interest included psychotherapy and first-line ADs. The selection of first-line ADs was guided by our preliminary findings regarding their usage. Among the study cohort of 101 953 beneficiaries, 46 104 (45.2%) did not receive any treatment, 14 881 (14.6%) received psychotherapy, and the most common first-line ADs included sertraline (9442 patients [9.2%]), escitalopram (9133 patients [9.0%]), citalopram (4760 patients [4.7%]), mirtazapine (3921 patients [3.8%]), duloxetine (3171 patients [3.1%]), trazodone (2978 [2.9%]), fluoxetine (2840 patients [2.8%]), bupropion (2326 patients [2.3%]), paroxetine (1389 patients [1.4%]), and venlafaxine (1028 patients [1.0%]).

### Outcome

Our outcome of interest was time to the first occurrence of FRI, including falls, bone fractures, sprains, strains, dislocations, and superficial skin injuries in the head, neck or trunk, upper limb, and lower limb. For our primary analysis, we identified FRI using the Acute Care Algorithm developed and validated by Min et al.^22^ This algorithm identified the most valid and severe FRI reported at hospitals or EDs to maximize the positive predictive value (PPV), which is positively associated with specificity and thereby reducing misclassification bias^21^ (PPV, 88.6%; sensitivity, 62.1%).^22^ While this algorithm initially used the *International Classification of Diseases, Ninth Revision *(*ICD-9*) codes, they have also developed an *International Statistical Classification of Diseases and Related Health Problems, Tenth Revision *(*ICD-10*) crosswalk for compatibility (eTable 2 in Supplement 1).

### Covariates

Baseline covariates were measured in the year prior to AD initiation, including sociodemographics (eg, age, sex, race), comorbidities (eg, anxiety disorder, bipolar disorder) (eTable 3 in Supplement 1),^23^ concomitant medication use (eg, antidementia agents, anxiolytics),^23^ health care use (eg, number of ED visits and outpatient visits), and clinician-related factors (eg, sex, specialty, average monthly prescribing fills and dose for ADs). Race and ethnicity were assessed due to their reported association with FRI risk,^24,25^ and they were identified from the master beneficiary summary files in the Medicare database, with categories defined based on Research Triangle Institute coding (American Indian and Alaskan Native, Asian or Pacific Islander, Black or African American, Hispanic, and non-Hispanic White). The medical conditions were considered present if a person had more than 1 diagnosis code for the condition, regardless of its position in any medical claims. We also calculated the Elixhauser comorbidity index to provide a comprehensive assessment of comorbidity.^26^ The use of a specific medication class was defined as having more than 1 fill for medication within that class. We also calculated the anticholinergic burden score as an index reflecting the extent of the anticholinergic adverse effects,^27^ and defined polypharmacy as the use of more than 5 different medications in total.^28^ The clinician was identified as the first physician who recorded the first diagnosis of depression in the patient’s medical records. The monthly prescribing dose for ADs was standardized using the defined daily dose launched by the World Health Organization.^29^

### Sensitivity Analyses

We performed a series of sensitivity analyses to ensure the robustness of the findings. First, we used a different algorithm (ie, inclusive algorithm; PPV, 75.2%; sensitivity, 68.4%) to maximize the inclusion of FRI events regardless of health care settings.^22^ Second, we shortened the follow-up window from 12 months to 6 months, considering FRI events often occur soon after treatment initiation. Third, we explored different grace period lengths (ie, 30 days), as the time from the initial depression diagnosis to the first depression treatment can vary across individuals in clinical settings.

### Statistical Analysis

Our primary goal was to evaluate FRI risk among older adults with depression across 11 first-line treatments and a control group receiving no treatment. We adopted the CCW approach, which enables us to emulate the valid analysis akin to a target clinical trial using observational data.^17,30^ First, we created clones for all eligible patients at baseline, assigning each clone to 1 of the 12 arms (ie, 11 treated and 1 control) on the index date, initiating follow-up. This step emulates the randomization process in the target trial, effectively eliminating confounding by baseline covariates. Given that clinical practice may not involve immediate treatment on the index date, we defined a grace period during which treatment initiation could occur. In the primary analysis, this grace period spanned 90 days, considering patients usually receive first-line treatments during the acute phase, defined as 84 to 114 days by the Health Care Effectiveness Data and Information Set measurement^31^ or 6 to 12 weeks according to UpToDate.^32^

Second, we censored any clone deviating from their assigned treatment during the grace period. For example, if a clone was assigned to fluoxetine on the index date but failed to initiate fluoxetine within the grace period while remaining in the cohort, the clone was considered treatment deviation at the end of the grace period and was censored at the time point. Other scenarios of treatment deviation are shown in eFigure 2 in Supplement 1. Considering this artificial censoring may introduce selection bias, we addressed it through inverse-probability-of-censoring weighting (IPCW).^33^ IPCW up-weights patients remaining in the risk set to compensate for censored patients, ensuring comparability between the 2 study groups throughout the grace period. We used a multivariable Cox proportional hazards regression to estimate the probability of an individual remaining uncensored at the end of the grace period, accounting for baseline covariates and estimating the censoring mechanism. The proportional hazards assumption was examined by visual assessment of the plotted Schoenfeld residuals.^34^

Finally, we accounted for IPCW in the Kaplan-Meier curve to estimate the per-protocol effect of individual first-line depression treatments on the 1-year FRI rate and restricted mean survival time (RMST). RMST represents the area under the survival curve up to a specific time point and can be interpreted as the mean time before the event or censoring points.^35^ The 95% CI for the FRI rate and RMST were derived from nonparametric bootstrap with 1000 replicates.^36^ In addition, we reported relative FRI rates and relative RMST using the approach proposed by Altman et al.^37^ We addressed the potential competing risks (ie, death and treatment switching, combination, or discontinuation) using the approaches in Calkins et al.^38^

We also reported the crude and adjusted hazard ratios (aHR) with 95% CI for the time to the first occurrence of FRI events associated with the exposure to a specific depression treatment compared with no treatment. Considering the potential competing risk, we used subdistribution hazard model instead of the conventional Cox proportional hazards model. Other details of the analysis were described in the eMethods and eFigure 2 in Supplement 1 illustrates different treatment scenario and treatment group assignments.

Baseline characteristics were described using mean (SD) for continuous variables and frequency and percentage for categorical variables. All analyses were performed using SAS version 9.4 (SAS Institute) between October 1, 2023, to March 31, 2024. Statistical significance was set at *P* < .05, and all tests were 2-sided.

## Results

As shown in Table 1, among 101 953 eligible beneficiaries (mean [SD] age, 76 [8] years), 63 344 (62.1%) were female, 7404 (7.3%) were Black individuals, and 81 856 (80.3%) were White individuals. The most prevalent comorbidities were hypertension (78 994 patients [77.5%]) and hyperlipidemia (73 665 patients [72.3%]), while the most prescribed comedications were lipid-modifying agents (47 839 patients [46.9%]) and ACEIs and ARBs (44 119 [43.3%]), aligning with the prevalent comorbidities. The rates of treatment change (ie, switching, combination, discontinuation) ranged from 22.9% to 62.6% and the death rate ranged from 0.5% to 3.4% across different treatment groups.

**Table 1.** Patient Characteristics of Eligible Medicare Beneficiaries With Depression: Overall and by Antidepressant

Characteristic	Patients, No. (%)												
	All	Control	Psychotherapy	Sertraline	Escitalopram	Citalopram	Mirtazapine	Duloxetine	Trazodone	Fluoxetine	Bupropion	Paroxetine	Venlafaxine
Total	101 953 (100.0)	46 104 (45.2)	14 881 (14.6)	9422 (9.2)	9133 (9.0)	4760 (4.7)	3921 (3.8)	3171 (3.1)	2978 (2.9)	2840 (2.8)	2326 (2.3)	1389 (1.4)	1028 (1.0)
Patient-level													
Age, mean (SD), y	75.9 (7.7)	75.8 (7.7)	75.5 (7.9)	76.6 (7.6)	76.2 (7.4)	76.2 (7.5)	79.8 (8.2)	74.8 (7.1)	76.2 (7.8)	74.7 (7.0)	72.6 (6.2)	75.9 (7.3)	73.9 (6.9)
Sex													
Female	63 344 (62.1)	27 968 (60.7)	9302 (62.5)	5965 (63.3)	5948 (65.1)	3138 (65.9)	2413 (61.5)	2064 (65.1)	1750 (58.8)	1803 (63.5)	1399 (60.1)	915 (65.9)	679 (66.0)
Male	38 609 (37.9)	18 136 (39.3)	5579 (37.5)	3457 (36.7)	3185 (34.9)	1622 (34.1)	1508 (38.5)	1107 (34.9)	1228 (41.2)	1037 (36.5)	927 (39.9)	474 (34.1)	349 (33.9)
Race													
American Indian or Alaskan Native	1634 (1.6)	775 (1.7)	298 (2.0)	122 (1.3)	121 (1.3)	55 (1.2)	40 (1.0)	57 (1.8)	50 (1.7)	40 (1.4)	40 (1.7)	18 (1.3)	18 (1.8)
Asian or Pacific Islander	2356 (2.3)	1239 (2.7)	221 (1.5)	194 (2.1)	170 (1.9)	73 (1.5)	176 (4.5)	47 (1.5)	95 (3.2)	62 (2.2)	22 (0.9)	36 (2.6)	21 (2.0)
Black	7404 (7.3)	3951 (8.6)	1066 (7.2)	508 (5.4)	387 (4.2)	257 (5.4)	393 (10.0)	192 (6.1)	254 (8.5)	154 (5.4)	111 (4.8)	78 (5.6)	53 (5.2)
White	81 856 (80.3)	35 915 (77.9)	12 328 (82.8)	7758 (82.3)	7697 (84.3)	3985 (83.7)	2912 (74.3)	2683 (84.6)	2245 (75.4)	2354 (82.9)	2029 (87.2)	1088 (78.3)	862 (83.9)
Others	671 (0.7)	327 (0.7)	92 (0.6)	64 (0.7)	63 (0.7)	32 (0.7)	26 (0.7)	15 (0.5)	<11^a^	<11^a^	13 (0.6)	13 (0.9)	<11^a^
Disability	1773 (1.7)	969 (2.1)	200 (1.3)	114 (1.2)	107 (1.2)	69 (1.4)	47 (1.2)	65 (2.0)	50 (1.7)	43 (1.5)	60 (2.6)	21 (1.5)	28 (2.7)
Dual eligibility	24 184 (23.7)	11 463 (24.9)	3575 (24.0)	2143 (22.7)	1637 (17.9)	1012 (21.3)	1295 (33.0)	646 (20.4)	875 (29.4)	614 (21.6)	358 (15.4)	368 (26.5)	198 (19.3)
Low-income subsidy	23 838 (23.4)	11 327 (24.6)	3303 (22.2)	2147 (22.8)	1633 (17.9)	1008 (21.2)	1296 (33.1)	667 (21.0)	864 (29.0)	631 (22.2)	376 (16.2)	380 (27.4)	206 (20.0)
Metropolitan	85 603 (84.0)	38 856 (84.3)	13 559 (91.1)	7526 (79.9)	7634 (83.6)	3619 (76.0)	3268 (83.3)	2578 (81.3)	2512 (84.4)	2195 (77.3)	1930 (83.0)	1109 (79.8)	817 (79.5)
Region													
Northeast	14 490 (14.2)	6752 (14.6)	2968 (19.9)	1263 (13.4)	1160 (12.7)	455 (9.6)	544 (13.9)	295 (9.3)	331 (11.1)	249 (8.8)	228 (9.8)	154 (11.1)	91 (8.9)
Midwest	16581 (16.3)	7317 (15.9)	2105 (14.1)	1874 (19.9)	1529 (16.7)	846 (17.8)	684 (17.4)	563 (17.8)	394 (13.2)	539 (19.0)	361 (15.5)	193 (13.9)	176 (17.1)
South	57 031 (55.9)	25 274 (54.8)	8104 (54.5)	5105 (54.2)	5371 (58.8)	2832 (59.5)	2181 (55.6)	1908 (60.2)	1794 (60.2)	1574 (55.4)	1396 (60.0)	878 (63.2)	614 (59.7)
West	13 851 (13.6)	6761 (14.7)	1704 (11.5)	1180 (12.5)	1073 (11.7)	627 (13.2)	512 (13.1)	405 (12.8)	459 (15.4)	478 (16.8)	341 (14.7)	164 (11.8)	147 (14.3)
Comorbidities													
Alcohol use disorder	2002 (2.0)	898 (1.9)	329 (2.2)	147 (1.6)	149 (1.6)	65 (1.4)	112 (2.9)	61 (1.9)	90 (3.0)	53 (1.9)	51 (2.2)	29 (2.1)	18 (1.8)
Anxiety disorder	16 821 (16.5)	6960 (15.1)	2788 (18.7)	1592 (16.9)	1573 (17.2)	807 (17.0)	657 (16.8)	566 (17.8)	593 (19.9)	439 (15.5)	356 (15.3)	308 (22.2)	182 (17.7)
Bipolar disorder	880 (0.9)	473 (1.0)	193 (1.3)	33 (0.4)	23 (0.3)	15 (0.3)	35 (0.9)	19 (0.6)	39 (1.3)	17 (0.6)	23 (1.0)	<11^a^	<11^a^
Chronic pain	10 835 (10.6)	4782 (10.4)	1521 (10.2)	900 (9.6)	815 (8.9)	454 (9.5)	415 (10.6)	763 (24.1)	397 (13.3)	279 (9.8)	259 (11.1)	119 (8.6)	131 (12.7)
Congestive heart failure	16 064 (15.8)	7692 (16.7)	2471 (16.6)	1411 (15.0)	1176 (12.9)	635 (13.3)	796 (20.3)	475 (15.0)	492 (16.5)	356 (12.5)	265 (11.4)	190 (13.7)	105 (10.2)
Dementia	10 869 (10.7)	4485 (9.7)	2033 (13.7)	1041 (11.0)	919 (10.1)	478 (10.0)	840 (21.4)	176 (5.6)	409 (13.7)	199 (7.0)	90 (3.9)	121 (8.7)	78 (7.6)
Diabetes	34 359 (33.7)	16 301 (35.4)	4740 (31.9)	3052 (32.4)	2820 (30.9)	1533 (32.2)	1303 (33.2)	1126 (35.5)	1018 (34.2)	949 (33.4)	734 (31.6)	459 (33.0)	324 (31.5)
Drug abuse	2115 (2.1)	947 (2.1)	314 (2.1)	151 (1.6)	124 (1.4)	88 (1.8)	89 (2.3)	137 (4.3)	114 (3.8)	49 (1.7)	48 (2.1)	32 (2.3)	22 (2.1)
Epilepsy	1865 (1.8)	878 (1.9)	353 (2.4)	152 (1.6)	134 (1.5)	79 (1.7)	93 (2.4)	37 (1.2)	66 (2.2)	33 (1.2)	11 (0.5)	14 (1.0)	15 (1.5)
Hyperlipidemia	73 665 (72.3)	33 169 (71.9)	10 805 (72.6)	6897 (73.2)	6740 (73.8)	3379 (71.0)	2740 (69.9)	2346 (74.0)	2178 (73.1)	1974 (69.5)	1701 (73.1)	1003 (72.2)	733 (71.3)
Hypertension	78 994 (77.5)	35 878 (77.8)	11 053 (74.3)	7411 (78.7)	7095 (77.7)	3710 (77.9)	3197 (81.5)	2563 (80.8)	2379 (79.9)	2160 (76.1)	1707 (73.4)	1092 (78.6)	749 (72.9)
Hypotension	5457 (5.4)	2368 (5.1)	1001 (6.7)	464 (4.9)	397 (4.3)	233 (4.9)	356 (9.1)	150 (4.7)	165 (5.5)	124 (4.4)	75 (3.2)	72 (5.2)	52 (5.1)
Ischemic heart disease	31 081 (30.5)	14 296 (31.0)	4631 (31.1)	2807 (29.8)	2613 (28.6)	1360 (28.6)	1341 (34.2)	1005 (31.7)	942 (31.6)	732 (25.8)	650 (27.9)	431 (31.0)	273 (26.6)
Osteoarthritis	34 548 (33.9)	15 602 (33.8)	5058 (34.0)	3088 (32.8)	3039 (33.3)	1538 (32.3)	1286 (32.8)	1526 (48.1)	1065 (35.8)	866 (30.5)	691 (29.7)	441 (31.7)	348 (33.9)
Osteoporosis	13 384 (13.1)	5911 (12.8)	2177 (14.6)	1218 (12.9)	1158 (12.7)	601 (12.6)	613 (15.6)	439 (13.8)	391 (13.1)	300 (10.6)	261 (11.2)	180 (13.0)	135 (13.1)
Parkinson disease	2078 (2.0)	866 (1.9)	376 (2.5)	206 (2.2)	178 (1.9)	84 (1.8)	118 (3.0)	41 (1.3)	74 (2.5)	38 (1.3)	36 (1.5)	38 (2.7)	23 (2.2)
Schizophrenia	581 (0.6)	335 (0.7)	125 (0.8)	17 (0.2)	14 (0.2)	11 (0.2)	29 (0.7)	<11^a^	24 (0.8)	<11^a^	<11^a^	<11^a^	<11^a^
Stroke	6675 (6.5)	2660 (5.8)	1393 (9.4)	647 (6.9)	560 (6.1)	305 (6.4)	332 (8.5)	166 (5.2)	217 (7.3)	188 (6.6)	93 (4.0)	67 (4.8)	47 (4.6)
Syncope	6035 (5.9)	2551 (5.5)	1054 (7.1)	557 (5.9)	534 (5.8)	277 (5.8)	318 (8.1)	170 (5.4)	195 (6.5)	151 (5.3)	97 (4.2)	79 (5.7)	52 (5.1)
Urinary incontinence	11 027 (10.8)	4777 (10.4)	1812 (12.2)	991 (10.5)	973 (10.7)	493 (10.4)	453 (11.6)	398 (12.6)	345 (11.6)	277 (9.8)	238 (10.2)	150 (10.8)	120 (11.7)
Vertigo	14 390 (14.1)	6098 (13.2)	2275 (15.3)	1411 (15.0)	1353 (14.8)	663 (13.9)	640 (16.3)	479 (15.1)	427 (14.3)	390 (13.7)	301 (12.9)	209 (15.0)	144 (14.0)
Vision disorder	57 286 (56.2)	25 503 (55.3)	8962 (60.2)	5300 (56.3)	5238 (57.4)	2672 (56.1)	2112 (53.9)	1807 (57.0)	1615 (54.2)	1490 (52.5)	1259 (54.1)	783 (56.4)	545 (53.0)
Elixhauser Comorbidity Index, mean (SD)	4.3 (3.2)	4.4 (3.2)	4.5 (3.4)	4.2 (3.0)	4.0 (3.0)	4.0 (3.0)	5.2 (3.3)	4.5 (3.0)	4.5 (3.2)	3.9 (2.9)	3.8 (2.8)	4.3 (3.1)	4.0 (3.0)
Comedications													
ACEIs/ARBs	44 119 (43.3)	19 847 (43.0)	5554 (37.3)	4373 (46.4)	4269 (46.7)	2153 (45.2)	1646 (42.0)	1497 (47.2)	1327 (44.6)	1303 (45.9)	1084 (46.6)	618 (44.5)	448 (43.6)
Antiarrhythmic agents (class 1 & 3)	2420 (2.4)	1145 (2.5)	349 (2.3)	234 (2.5)	188 (2.1)	64 (1.3)	118 (3.0)	68 (2.1)	66 (2.2)	63 (2.2)	65 (2.8)	30 (2.2)	30 (2.9)
Anticonvulsants	12 665 (12.4)	5543 (12.0)	1780 (12.0)	1150 (12.2)	1014 (11.1)	577 (12.1)	481 (12.3)	779 (24.6)	459 (15.4)	313 (11.0)	264 (11.3)	159 (11.4)	146 (14.2)
Antidementia agents	5355 (5.3)	1987 (4.3)	790 (5.3)	685 (7.3)	589 (6.4)	305 (6.4)	394 (10.0)	93 (2.9)	207 (7.0)	122 (4.3)	63 (2.7)	70 (5.0)	50 (4.9)
Antidiabetics	19 669 (19.3)	9286 (20.1)	2433 (16.3)	1871 (19.9)	1663 (18.2)	981 (20.6)	689 (17.6)	678 (21.4)	582 (19.5)	594 (20.9)	461 (19.8)	247 (17.8)	184 (17.9)
Antihistamines	1590 (1.6)	722 (1.6)	160 (1.1)	150 (1.6)	150 (1.6)	87 (1.8)	80 (2.0)	58 (1.8)	58 (1.9)	51 (1.8)	38 (1.6)	22 (1.6)	14 (1.4)
Antiparkinson agents	2851 (2.8)	1206 (2.6)	427 (2.9)	290 (3.1)	239 (2.6)	140 (2.9)	114 (2.9)	93 (2.9)	116 (3.9)	83 (2.9)	66 (2.8)	46 (3.3)	31 (3.0)
Antipsychotics	2826 (2.8)	1472 (3.2)	486 (3.3)	177 (1.9)	156 (1.7)	72 (1.5)	177 (4.5)	32 (1.0)	134 (4.5)	39 (1.4)	39 (1.7)	20 (1.4)	22 (2.1)
Antithrombotics	17 524 (17.2)	8057 (17.5)	2393 (16.1)	1746 (18.5)	1525 (16.7)	778 (16.3)	798 (20.4)	563 (17.8)	507 (17.0)	448 (15.8)	341 (14.7)	225 (16.2)	143 (13.9)
Anxiolytics	10 517 (10.3)	3878 (8.4)	1598 (10.7)	1103 (11.7)	1201 (13.2)	565 (11.9)	440 (11.2)	384 (12.1)	413 (13.9)	327 (11.5)	246 (10.6)	225 (16.2)	137 (13.3)
Beta blockers	35 138 (34.5)	16 180 (35.1)	4636 (31.2)	3406 (36.1)	3158 (34.6)	1608 (33.8)	1448 (36.9)	1130 (35.6)	1056 (35.5)	978 (34.4)	737 (31.7)	478 (34.4)	323 (31.4)
Bisphosphonates	440 (0.4)	190 (0.4)	71 (0.5)	51 (0.5)	32 (0.4)	22 (0.5)	16 (0.4)	18 (0.6)	<11^a^	16 (0.6)	<11^a^	<11^a^	<11^a^
CCB	23 593 (23.1)	10 749 (23.3)	3000 (20.2)	2273 (24.1)	2139 (23.4)	1125 (23.6)	1060 (27.0)	767 (24.2)	766 (25.7)	616 (21.7)	529 (22.7)	353 (25.4)	216 (21.0)
Diuretics	23 520 (23.1)	10 889 (23.6)	3078 (20.7)	2276 (24.2)	1976 (21.6)	1110 (23.3)	951 (24.3)	798 (25.2)	692 (23.2)	668 (23.5)	551 (23.7)	317 (22.8)	214 (20.8)
Hypnotics/sedatives	4289 (4.2)	1544 (3.3)	667 (4.5)	420 (4.5)	484 (5.3)	212 (4.5)	174 (4.4)	179 (5.6)	251 (8.4)	104 (3.7)	130 (5.6)	69 (5.0)	55 (5.4)
Lipid-modifying agents	47 839 (46.9)	21 364 (46.3)	6593 (44.3)	4715 (50.0)	4487 (49.1)	2279 (47.9)	1759 (44.9)	1564 (49.3)	1435 (48.2)	1345 (47.4)	1156 (49.7)	680 (49.0)	462 (44.9)
Mood stabilizers	1018 (1.0)	533 (1.2)	182 (1.2)	65 (0.7)	47 (0.5)	25 (0.5)	54 (1.4)	20 (0.6)	48 (1.6)	15 (0.5)	17 (0.7)	<11^a^	<11^a^
Muscle relaxants	2387 (2.3)	1070 (2.3)	296 (2.0)	194 (2.1)	202 (2.2)	90 (1.9)	73 (1.9)	176 (5.6)	86 (2.9)	66 (2.3)	58 (2.5)	39 (2.8)	37 (3.6)
NSAIDs	7950 (7.8)	3440 (7.5)	965 (6.5)	725 (7.7)	785 (8.6)	411 (8.6)	234 (6.0)	472 (14.9)	241 (8.1)	265 (9.3)	190 (8.2)	124 (8.9)	98 (9.5)
Vasodilators	3973 (3.9)	1945 (4.2)	445 (3.0)	356 (3.8)	342 (3.7)	190 (4.0)	185 (4.7)	136 (4.3)	117 (3.9)	94 (3.3)	68 (2.9)	62 (4.5)	33 (3.2)
Opioids	11 624 (11.4)	5192 (11.3)	1259 (8.5)	994 (10.5)	886 (9.7)	524 (11.0)	499 (12.7)	901 (28.4)	461 (15.5)	318 (11.2)	290 (12.5)	156 (11.2)	144 (14.0)
Other antihypertensives	4733 (4.6)	2209 (4.8)	612 (4.1)	482 (5.1)	429 (4.7)	219 (4.6)	212 (5.4)	141 (4.4)	160 (5.4)	95 (3.3)	83 (3.6)	51 (3.7)	40 (3.9)
Parasympathomimetics	260 (0.3)	115 (0.2)	32 (0.2)	26 (0.3)	28 (0.3)	11 (0.2)	15 (0.4)	13 (0.4)	<11^a^	<11^a^	<11^a^	<11^a^	<11^a^
Psychostimulants	359 (0.4)	155 (0.3)	72 (0.5)	22 (0.2)	28 (0.3)	13 (0.3)	<11^a^	15 (0.5)	<11^a^	<11^a^	20 (0.9)	<11^a^	<11^a^
Systemic steroids	4742 (4.7)	2128 (4.6)	585 (3.9)	445 (4.7)	423 (4.6)	226 (4.7)	231 (5.9)	208 (6.6)	152 (5.1)	127 (4.5)	106 (4.6)	73 (5.3)	38 (3.7)
Anticholinergic burden index, mean (SD)	1.0 (1.7)	1.0 (1.7)	1.0 (1.6)	1.6 (1.8)	0.9 (1.6)	1.0 (1.6)	1.2 (1.8)	1.2 (1.8)	1.2 (1.8)	1.0 (1.7)	0.9 (1.5)	1.5 (1.8)	0.9 (1.5)
Polypharmacy	11 643 (11.4)	5311 (11.5)	1480 (9.9)	1112 (11.8)	966 (10.6)	528 (11.1)	489 (12.5)	504 (15.9)	457 (15.3)	288 (10.1)	229 (9.8)	166 (12.0)	113 (11.0)
Health care utilization, mean (SD)													
Outpatient visits	6.6 (9.8)	6.6 (9.9)	6.1 (9.2)	7.0 (9.9)	6.1 (9.1)	6.7 (9.7)	8.1 (11.6)	8.1 (10.9)	6.8 (10.3)	6.6 (9.7)	6.1 (8.7)	6.2 (9.4)	7.4 (10.4)
ED visits	0.7 (1.4)	0.7 (1.4)	0.8 (1.5)	0.7 (1.4)	0.7 (1.3)	0.7 (1.3)	1.0 (1.6)	0.8 (1.6)	0.8 (1.5)	0.7 (1.4)	0.5 (1.1)	0.7 (1.3)	0.7 (1.7)
Total length of hospitalization	2.1 (6.6)	2.0 (6.5)	3.2 (8.3)	1.8 (5.8)	1.6 (5.8)	1.8 (6.3)	3.2 (8.1)	1.7 (5.4)	2.3 (7.0)	1.8 (5.6)	1.2 (4.4)	1.7 (4.8)	1.4 (4.6)
Clinician-level^b^													
Male	76 246 (74.8)	34 463 (74.8)	12 016 (80.7)	6759 (71.7)	6704 (73.4)	3439 (72.2)	2875 (73.3)	2314 (73.0)	2237 (75.1)	2000 (70.4)	1655 (71.2)	1045 (75.2)	739 (71.9)
Monthly patients receiving AD, monthly, mean (SD)	0.5 (1.2)	0.6 (1.3)	0.5 (1.3)	0.4 (0.9)	0.5 (1.0)	0.5 (1.0)	0.7 (1.8)	0.5 (1.1)	0.6 (1.5)	0.4 (0.9)	0.4 (0.9)	0.5 (0.9)	0.4 (0.7)
Monthly AD fills, mean (SD)	2.3 (7.4)	2.4 (7.4)	2.4 (8.1)	1.9 (5.9)	2.1 (6.1)	2.1 (6.8)	3.2 (12.1)	2.3 (6.2)	2.7 (9.7)	1.7 (5.5)	2.0 (5.4)	2.1 (5.0)	1.8 (3.8)
Monthly AD dose, mean (SD)	0.7 (1.7)	0.6 (1.6)	0.5 (1.9)	0.7 (1.5)	0.8 (1.8)	0.8 (1.5)	0.8 (1.9)	0.8 (1.8)	0.8 (2.0)	0.6 (1.2)	0.9 (1.8)	0.7 (1.3)	0.8 (1.5)
Specialty													
General internal medicine	51 803 (50.8)	23 434 (50.8)	4055 (27.2)	5797 (61.5)	5496 (60.2)	2998 (63.0)	2071 (52.8)	1788 (56.4)	1531 (51.4)	1797 (63.3)	1423 (61.2)	819 (59.0)	594 (57.8)
Psychiatry	1159 (1.1)	355 (0.8)	618 (4.2)	38 (0.4)	33 (0.4)	15 (0.3)	37 (0.9)	13 (0.4)	22 (0.7)	16 (0.6)	<11^a^	<11^a^	<11^a^
Others	48 991 (48.1)	22 315 (48.4)	10 208 (68.6)	3587 (38.1)	3604 (39.5)	1747 (36.7)	1813 (46.2)	1370 (43.2)	1425 (47.9)	1027 (36.2)	895 (38.5)	569 (41.0)	431 (41.9)
FRI events	6310 (6.2)	2877 (6.2)	1093 (7.3)	490 (5.2)	479 (5.2)	257 (5.4)	269 (6.9)	198 (6.2)	230 (7.7)	135 (4.8)	69 (3.0)	65 (4.7)	66 (6.4)
Competing events													
Treatment change	25 816 (46.2)	NA	3402 (22.9)	5579 (59.2)	5352 (58.6)	2827 (59.4)	1572 (40.1)	1497 (47.2)	865 (29.0)	1607 (56.6)	1455 (62.6)	756 (54.4)	575 (55.9)
Death	2077 (2.0)	1556 (3.4)	172 (1.2)	66 (0.7)	59 (0.6)	25 (0.5)	86 (2.2)	19 (0.6)	28 (0.9)	21 (0.7)	14 (0.6)	15 (1.1)	<11^a^

Abbreviations: ACEI, angiotensin converting enzyme inhibitor; AD, antidepressants; ARB, angiotensin receptor blocker; CCB, calcium channel blocker; ED, emergency department; FRI, falls and related injuries; NA, not applicable; NSAID, nonsteroidal anti-inflammatory drug.

^a^ The Centers for Medicare & Medicaid Services mandates that data representing small sample sizes, typically those with fewer than 11 observations, be suppressed or masked in reporting to maintain patient confidentiality.

^b^ The clinician was identified as the first physician who recorded the diagnosis of depression in the patient’s medical records. The missingness of the clinician-related factors was 22.1%.

**Figure 1.**
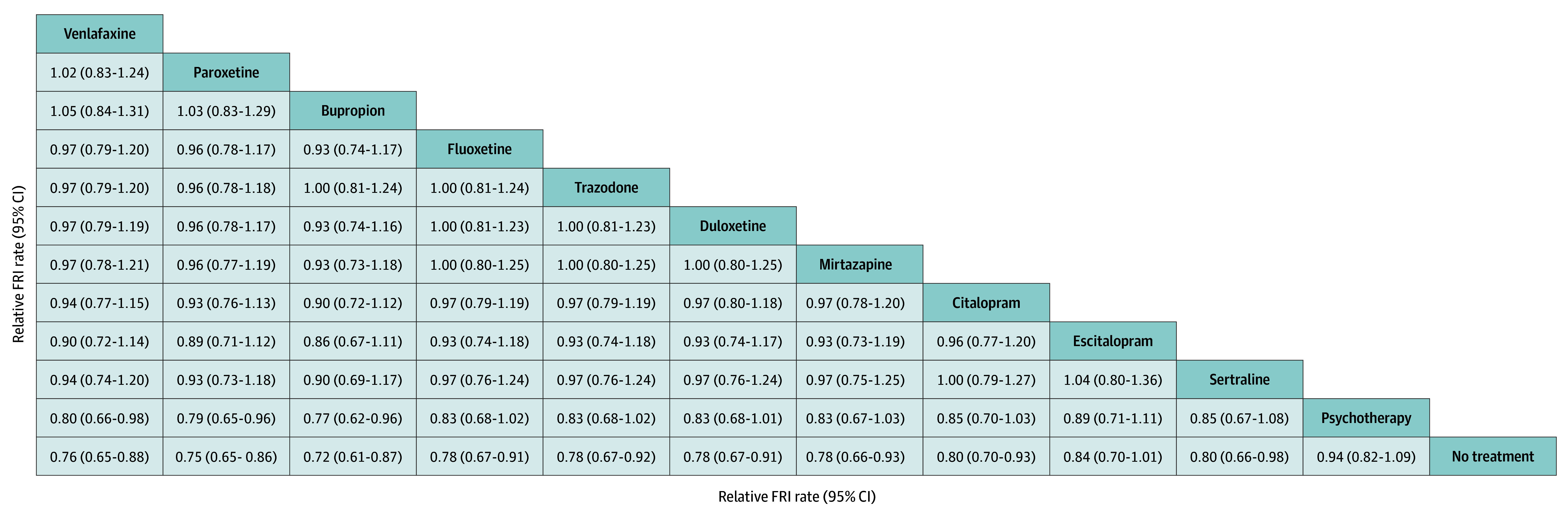
Relative FRI Rates in the Main Analysis Falls and related injuries (FRI) was the acute care algorithm, the grace period was 90 days, and follow-up was 365 days.

**Figure 2.**
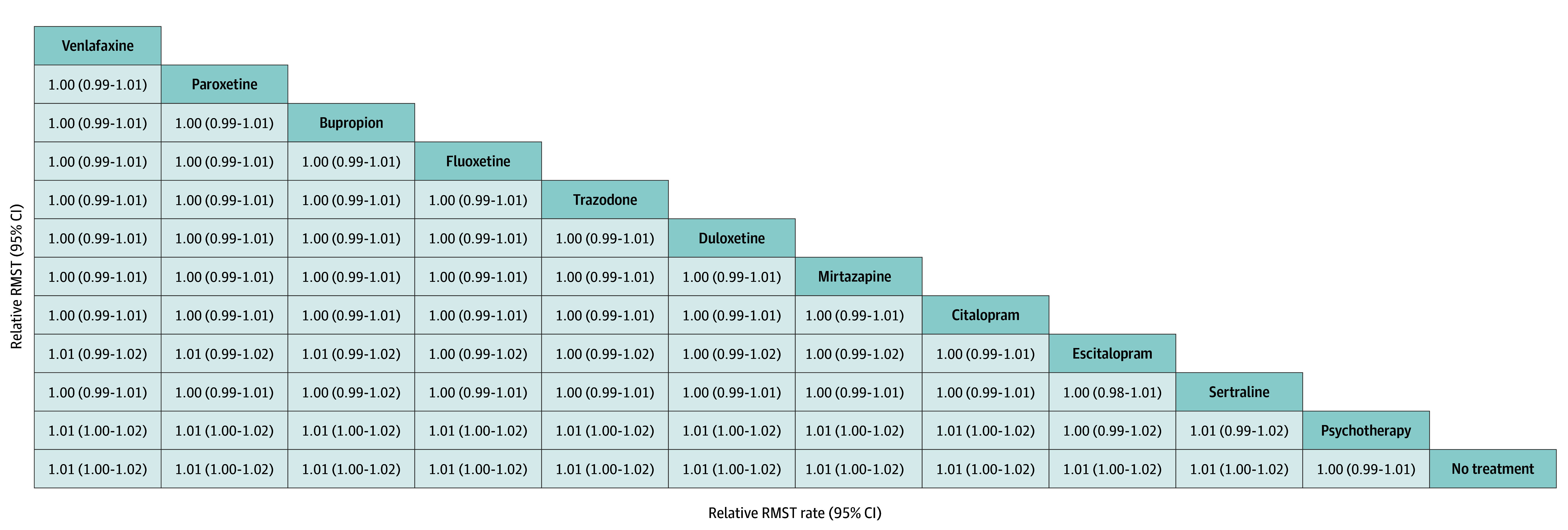
Relative RMST in the Main Analysis Falls and related injuries (FRI) was the acute care algorithm, the grace period was 90 days, and follow-up was 365 days. RMST indicates restricted mean survival time.

Compared with the control group (ie, no treatment), psychotherapy was not associated with FRI risk (adjusted HR, 0.94; 95% CI, 0.82-1.17), while first-line ADs were associated with a decreased FRI risk (adjusted HR ranged from 0.74 [95% CI, 0.59-0.89] for bupropion to 0.83 [95% CI, 0.67-0.98] for escitalopram). The FRI event rate was 87 (95% CI, 83-90) per 1000 person-year in the control group, ranging from 63 (95% CI, 53-75) per 1000 person-year for buproprion to 82 (95% CI, 74-97) per 1000 person-year for psychotherapy in the treated groups. The RMST was 349 (95% CI, 346-350) days in the control group and ranged from 350 (95% CI, 346-352) days for psychotherapy to 353 (95% CI, 350-356) days for fluoxetine and bupropion in the treated groups (Table 2). Similar to the adjusted HR, the relative FRI rates suggested that while psychotherapy may not significantly affect the FRI rate (0.94; 95% CI, 0.82-1.09), first-line ADs were associated with a decreased FRI risk (ranging from buproprion [FRI rate, 0.72; 95% CI, 0.61-0.87] to sertraline [FRI rate, 0.80; 95% CI, 0.66-0.98]) (Figure 1). By contrast, the relative RMST between individual first-line depression treatments indicated no significant difference in the associated FRI risk (RMST, 1.00; 95% CI, 0.99-1.02) (Figure 2).

**Table 2.** FRI Rate, RMST, and HR^a^

Treatment	Event rate (95% CI), per 1000 person-year	RMST (95% CI), d	HR (95% CI)
No treatment	87 (83-90)	349 (346-350)	1 [Reference]
Psychotherapy	82 (74-97)	350 (346-352)	0.94 (0.82-1.17)
Sertraline	70 (57-84)	352 (348-355)	0.81 (0.63-0.95)
Escitalopram	73 (58-83)	351 (348-355)	0.83 (0.67-0.98)
Citalopram	70 (61-80)	352 (349-354)	0.81 (0.69-0.92)
Mirtazapine	68 (58-81)	352 (350-355)	0.78 (0.65-0.96)
Duloxetine	68 (60-80)	352 (349-354)	0.78 (0.68-0.91)
Trazodone	68 (56-76)	353 (349-355)	0.78 (0.62-0.90)
Fluoxetine	68 (57-77)	353 (350-356)	0.78 (0.65-0.91)
Bupropion	63 (53-75)	353 (350-356)	0.74 (0.59-0.89)
Paroxetine	65 (59-78)	353 (349-355)	0.74 (0.66-0.92)
Venlafaxine	66 (57-76)	353 (345-356)	0.75 (0.64-0.90)

Abbreviations: FRI, fall and related injuries; HR, hazard ratio; RMST, restricted mean survival time.

^a^ FRI was acute care algorithm, grace period of 90 days, and follow-up of 365 days.

The overall findings were generally consistent across different sensitivity analyses. The FRI incidence was approximately 2.5 times higher and RMST decreased with the inclusive algorithm, which captured FRI in all health care settings, compared with the acute care algorithm, which only include FRIs from inpatient and ED settings (eTable 4 in Supplement 1). Adjusted HRs were similar to the main analysis, but the 95% CIs were more narrow due to more events. Shortening the follow-up period from 365 to 183 days halved FRI incidence and RMST, with similar adjusted HRs and 95% CIs to the main analysis (eTable 5 in Supplement 1). Furthermore, shortening the grace period from 90 to 30 days resulted in similar FRI incidence and RMST but lower adjusted HRs, suggesting that early treatment with antidepressants after initial depression diagnosis may improve depression and reduce FRI risks (eTable 6 in Supplement 1).

## Discussion

Using the TTE framework in conjunction with the CCW approach, we leveraged the Medicare administrative claims data from clinical practice to emulate a pragmatic clinical trial comparing no treatment with 11 first-line depression treatments (ie, psychotherapy, sertraline, escitalopram, citalopram, mirtazapine, duloxetine, trazodone, fluoxetine, bupropion, paroxetine, and venlafaxine). Our findings showed that, compared with no treatment, psychotherapy was not associated with FRI risk, while first-line ADs were associated with a decreased FRI risk.

Our findings contrast with previous studies, suggesting that AD use, compared with no use, may increase FRI risk due to their anticholinergic adverse effects, such as orthostatic hypotension, sedation, and syncope.^39^ Potential reasons for these different findings include differences in study populations (eg, previous studies included all older adults, while we focused on older adults with depression); designs subjected to confounding by indication, immortal bias, and competing risk; and less comprehensive comparisons of single treatments. By contrast, our findings aligned with studies taking the confounding by indication into consideration. For instance, a self-controlled case series study showed that the incidence rate ratio of hip fracture was the highest 16 to 30 days before AD initiation, indicating that hip fractures were highly likely attributed to depression rather than AD use.^40^ Additionally, a mediation analysis found that AD use accounted for only 18% of the association between depression and FRI.^41^ This evidence implies that the majority of this association resulted from depressive conditions and other factors.^41^ We observed a decreased FRI rate and RMST in patients receiving ADs compared with the untreated individuals, indicating that depression itself could be the main contributor to FRI. Consequently, this supports the hypothesis that the clinical benefits of ADs in managing depression may outweigh the risks associated with their anticholinergic adverse effects that contribute to FRI.

Notably, our study found that 45.2% of older adults diagnosed with depression did not receive psychotherapy or AD within the first 90 days of diagnosis. This aligns with a review^42^ highlighting that only 4% to 37% of elderly patients with depression received treatment within 2 years of diagnosis, leading to a majority experiencing a poor prognosis (only 33% attained wellness). These findings underscore the critical consequences of untreated depression, which can lead to cognitive decline, increased suicide risk, higher mortality rates, and reduced quality of life.^43^ ADs and psychotherapy, the mainstays of depression treatment, have been proven to significantly alleviate depressive symptoms.^42^ Although there have been concerns about FRI risk associated with these treatments in older adults, our findings suggest that standard first-line depression treatments do not exacerbate FRI risk, indicating their safety for initiation in older adults newly diagnosed with depression. However, it remains crucial for clinicians to consider the full spectrum of potential adverse effects and customize treatment plans to ensure a balance between effectiveness and risks.

### Limitations

This study has limitations. First, the specific definition of FRI may affect the evaluation of FRI risk. Second, the time elapsed from depression diagnosis to the initiation of the first treatment (ie, grace period) may vary among individuals. Third, short-term FRI risk may differ from long-term FRI risk. To account for these concerns, we performed several sensitivity analyses by varying the definition of FRI, the length of the follow-up window, and the length of the grace period, respectively, and our conclusions remained consistent. Fourth, the potential influence of confounding by patients’ underlying characteristics cannot be ruled out entirely. However, the CCW approach mimics randomization in clinical trials, which mitigates confounding. Fifth, FRI that did not receive medical attention were difficult to capture in claims data, potentially underestimating FRI incidence in our study. This nondifferential outcome misclassification may lead to bias toward the null. Regardless, we still observed significantly decreased risk of FRI in the patients receiving ADs compared with the untreated group. Sixth, we calculated time-fixed IPCW instead of time-varying IPCW because the covariates included, such as demographics, chronic comorbidities, chronic comedication use, are relatively stable and unlikely to change substantially within the follow-up period. This approach was chosen to manage the complexity of our analysis without compromising the validity of the results. Seventh, we acknowledged unmeasured confounders, such as lifestyle and living environment, that could influence the outcomes. Eighth, we did not consider the nonlinear relationship and the interaction of the covariates with treatment deviation while estimating IPCW. Lastly, we did not explore the impact of adjunctive or add-on therapies or switching to another AD or psychotherapy on FRI risk, nor did we examine the dose-response relationship. Investigating these clinically relevant aspects will be an important area of focus in future research endeavors.

## Conclusions

Our study suggests that first-line ADs were associated with a decreased FRI risk compared with untreated individuals. These findings provide valuable insights into the safety profiles of these treatments, aiding clinicians in their consideration for treating depression in older adults.

## Notes

Supplement 1. **eMethods. **Detailed Steps of the Cloning-Censoring-Weighting Approach **eTable 1. **Specification and Emulation of the Target Trial **eTable 2. **International Classification of Diseases Clinical Modification Codes for Fall and Related Injuries **eTable 3.** International Classification of Diseases Clinical Modification Codes for Comorbidities **eTable 4. **FRI Rates, RMST, and HRs for Sensitivity Analysis (Grace Period of 90 Days; Follow-Up of 365 Days) **eTable 5. **FRI Rate, RMST, and HRs for Sensitivity Analysis (Grace Period of 90 Days; Follow-Up of 183 Days) **eTable 6. **FRI Rates, RMST, and HRs for Sensitivity Analysis (Grace Period of 30 Days, Follow-Up of 365 Days) **eFigure 1. **Study Design Schematic Diagram **eFigure 2. **Treatment Scenarios Assignment, Censoring Weighting and Outcome Analysis in the Targeted Trial Emulation Approach

Supplement 2.
Data Sharing Statement
