# Supplementary material for: Injurious Fall Risk Differences Among Older Adults With First-Line Depression Treatments
Source: JAMA Netw Open. 2024 Aug 26;7(8):e2435535. doi: 10.1001/jamanetworkopen.2024.35535 (PMC12068830; doi:10.1001/jamanetworkopen.2024.35535)
Supplement: Supplement 1. — eMethods. Detailed Steps of the Cloning-Censoring-Weighting Approach eTable 1. Specification and Emulation of the Target Trial eTable 2. International Classification of Diseases Clinical Modification Codes for Fall and Related Injuries eTable 3. International Classification of Diseases Clinical Modification Codes for Comorbidities eTable 4. FRI Rates, RMST, and HRs for Sensitivity Analysis (Grace Period of 90 Days; Follow-Up of 365 Days) eTable 5. FRI Rate, RMST, and HRs for Sensitivity Analysis (Grace Period of 90 Days; Follow-Up of 183 Days) eTable 6. FRI Rates, RMST, and HRs for Sensitivity Analysis (Grace Period of 30 Days, Follow-Up of 365 Days) eFigure 1. Study Design Schematic Diagram eFigure 2. Treatment Scenarios Assignment, Censoring Weighting and Outcome Analysis in the Targeted Trial Emulation Approach [file jamanetwopen-e2435535-s001.pdf]

Supplemental Online Content

Wang GHM, Lai ECC, Goodin AJ, et al. Injurious fall risk differences among older adults with first-line depression treatments. *JAMA Netw Open*. 2024;7(8)e2435535:. doi:10.1001/jamanetworkopen.2024.35535

eMethods. Detailed Steps of the Cloning-Censoring-Weighting Approach

eTable 1. Specification and Emulation of the Target Trial

eTable 2. International Classification of Diseases Clinical Modification Codes for Fall and Related Injuries

eTable 3. International Classification of Diseases Clinical Modification Codes for Comorbidities

eTable 4. FRI Rates, RMST, and HRs for Sensitivity Analysis (Grace Period of 90 Days; Follow-Up of 365 Days)

eTable 5. FRI Rate, RMST, and HRs for Sensitivity Analysis (Grace Period of 90 Days; Follow-Up of 183 Days)

eTable 6. FRI Rates, RMST, and HRs for Sensitivity Analysis (Grace Period of 30 Days, Follow-Up of 365 Days)

eFigure 1. Study Design Schematic Diagram

eFigure 2. Treatment Scenarios Assignment, Censoring Weighting and Outcome Analysis in the Targeted Trial Emulation Approach

This supplemental material has been provided by the authors to give readers additional information about their work.

© 2024 Wang GHM et al. *JAMA Network Open.*

eMethods. Detailed steps of the cloning-censoring-weighting approach

Our primary goal was to evaluate FRI risk among older adults with depression across 11 first-line treatments and a control group receiving no treatment. We adopted the CCW approach which enables us to emulate the valid analysis akin to a target clinical trial using observational data.[1,2] First, we created clones for all eligible patients at baseline, assigning each clone to one of the 12 arms (i.e., 11 treated and one control) on the index date, initiating follow-up. This step emulates the randomization process in the target trial, effectively eliminating confounding by baseline covariates. Given that real-world clinical practice may not involve immediate treatment on the index date, which may cause immortal-time bias (i.e., patients have to stay alive and event-free until treatment initiation), we defined a grace period during which treatment initiation could occur. In the primary analysis, this grace period spanned 90 days considering patients usually receive first-line treatments during the acute phase, defined as 84-114 days by the Healthcare Effectiveness Data and Information Set measurement[3] or 6-12 weeks according to UpToDate.[4]

Second, we censored any clone deviating from their assigned treatment during the grace period. Considering this artificial censoring may introduce selection bias, we addressed it through inverse-probability-of-censoring weighting (IPCW). [5] IPCW up-weights patients remaining in the risk set to compensate for censored patients, ensuring comparability between the two arms throughout the grace period. We used a multivariable Cox proportional hazards regression model to predict the probability of an individual remaining uncensored at the end of the grace period, accounting for baseline covariates predictive of the censoring mechanism. We assumed all the covariates were linearly correlated with treatment deviation while estimating IPCW. This Cox model, referred to as the "censoring-weighting" model, met the proportional hazards assumptions, which was examined by visually assessing the plotted Schoenfeld residuals.[6] To calculate the probability of an individual remaining uncensored using the Cox model, we first built a baseline survival function with all covariates set to zero. Then, by applying each individual's covariate values, we obtained their specific survival function, from which we calculated the probability of remaining uncensored up to time T.

Finally, we accounted for IPCW in the Kaplan-Meier (K-M) curve to estimate the per-protocol effect of individual first-line depression treatments on the 1-year FRI rate and restricted mean survival time (RMST). The K-M curve was referred to as the "outcome

analysis” model in the following context. We addressed the potential competing risks, including treatment switching/combination/discontinuation and death, using the approaches mentioned in Calkins et al[7]. To address uncertainty in weight estimation and sample size inflation, we employed non-parametric bootstrap with 1000 replicates to derive the 95% CI for the FRI rate and RMST.[5] RMST is an alternative estimate for analyzing time-to-event data, representing the area under the survival curve up to a specific time point, and can be interpreted as the average time before the event or censoring points.[8] RMST provides a clinically meaningful way to interpret survival differences between two arms and has advantages over the conventionally used Cox model, as it does not rely on the proportional hazards assumption and it provides information on absolute risk.[9] We addressed potential competing risks, including treatment switching/combination/discontinuation and death, using the approaches mentioned in Calkins et al[7]. In addition, we reported relative FRI rates and relative RMST using the approach proposed by Altman et al.[10] Considering the potential competing risk, we used subdistribution hazard model instead of the conventional Cox proportional hazards model for estimating the crude and adjusted hazard ratios (aHR) with 95% CI for the time to the first occurrence of FRI events associated with the exposure to a specific depression treatment compared to no treatment.

As illustrated in eFigure 2, when a patient initiated a treatment (A) within the grace period (scenarios 1 to 4), then the “other treatments” and “untreated” clones were considered as having treatment deviation (δ_D=1) and were censored at the time of treatment A initiation (T_A) in the “cloning-censoring” model. The “other treatments” or “untreated” clones were thus considered as censored (δ_O=0) and the follow-up ended at T_A in the “outcome analysis” model. For the “treatment A” clones, they did not experience treatment deviation (δ_D=0), and we assigned T_A to them as the censoring time in the “censoring-weighting” model to allow the immortal time to contribute to all arms, thereby eliminating the immortal-time bias. The “treatment A” clones were then followed until the earliest of outcome (i.e., FRI) or censoring events (i.e., death, transition to Medicare Advantage plans, hospice services or nursing facility utilization, treatment switching/combination/discontinuation, or end of 1-year), and the event and time in the “outcome analysis” model were recorded based on whether or not the outcome occurred and the time to outcome or censoring events.

When a patient did not initiate any treatment within the grace period (scenarios 5 to 8), then the “treatment A” and “other treatments” clones were considered to have treatment

© 2024 Wang GHM et al. *JAMA Network Open.*

deviation (δ_D=1) and censored at the end of the grace period (T_G). The “treatment A” and “other treatments” clones were thus considered as censored (δ_O=0) and the follow-up ended at T_G in the “outcome analysis” model. For the “untreated” clones, they did not experience treatment deviation (δ_D=0), and we assigned T_G to them as the censoring time in the “censoring-weighting” model to allow the immortal time to contribute to all arms, thereby eliminating the immortal-time bias. The “untreated” clones were then followed until the earliest of outcome or administrative censoring, and the event and time in the “outcome analysis” model were recorded based on whether or not the outcome occurred and the time to outcome or censoring events.

If a clone experienced the outcome or censoring events before the end of grace period (scenarios 9 and 10), then all arms in these scenarios were considered as no treatment deviation (δ_D=0) because we did not observe it, and they were censored at the time of outcome or censoring event in the “cloning-weighting” model. For patients initiating treatments on the index date (scenarios 11 and 12), they were not cloned and were included in the analysis as an individual record. For all arms, the event and time in the “outcome analysis” model were recorded based on whether or not the outcome occurred and the time to outcome or censoring events.

© 2024 Wang GHM et al. *JAMA Network Open.*

eTable 1. Specification and Emulation of the Target Trial

Protocol component	Target trial	Emulation using Medicare data
Aim	To assess the association between specific first-line depression treatments and the FRI risk among older adults newly diagnosed with depression	Same
Eligibility criteria	Inclusion:<br>• Aged ≥65 years<br>• Diagnosed with depression (first diagnosis date=index date)<br>• Enrollment period: 2017 to 2018<br>Exclusion:<br>• Without continuous enrollment in the healthcare system of interest in the past year;<br>• Diagnosed with depression or received any psychotherapy/AD in the past year<br>• Had FRI in the past year<br>• Received hospice services or were in a nursing facility in the past year	Same
Treatment strategies	Psychotherapy and first-line ADs including sertraline, escitalopram, citalopram, mirtazapine, duloxetine, trazodone, fluoxetine, bupropion, paroxetine, and venlafaxine	Same, but we allowed a 90-day grace period between the index date and the 1st AD prescription date in the real world.
Assignment procedures	Eligible patients are randomly assigned to one or more treatment groups on the index date and are aware of the strategy they are assigned to.	Same, but we excluded patients initiating > 1 treatments on the 1st AD prescription date. Patients in each treatment group were assumed to be comparable after the CCW approach.
Follow-up	• Starts at treatment assignment<br>• Ends at the earliest of: FRI, death, switching to Medicare Advantage, utilization of hospice services or nursing facility, or end of follow-up	Same. We also censored patients switching to/combining other treatments, or treatment discontinuation.
Outcome	Time to first FRI	Same
Causal contrasts	Intention-to-treat effect<br>Per-protocol effect	Only per-protocol effect since a patient could be assigned to multiple groups on the index date.
Analysis plan	HR, 1-year FRI rate, and RMST between groups	Same

Abbreviations: AD: antidepressant, CCW: cloning-censoring-weighting, FRI: fall and related injuries, HR, hazard ratio, RMST: restricted mean survival time

© 2024 Wang GHM et al. *JAMA Network Open.*

eTable 2 International Classification of Diseases Clinical Modification Codes for Fall and Related Injuries

Type of FRI	ICD-10-CM
Falls	W010XX, W0110X, W01110, W01118, W01119, W01190, W01198, W050XX, W051XX, W052XX, W06XXX, W07XXX, W08XXX, W100XX, W101XX, W102XX, W108XX, W109XX, W1781X, W1789X, W1800X, W1802X, W1809X, W1812X, W182XX, W1839X, W1849X, W19XXX
Head/Face Injuries	S0190, S020X, S0210, S0211, S0219, S022X, S0230, S0231, S0232, S0240, S0241, S0242, S0260, S0261, S0262, S0263, S0264, S0265, S0266, S0267, S0269, S0280, S0281, S0282, S0291, S0292, S060X, S061X, S062X, S0630, S0631, S0632, S0633, S0634, S0635, S0636, S0637, S0638, S064X, S065X, S066X, S0681, S0682, S0689, S069X, S0000, S0001, S0003, S0010, S0011, S0012, S0020, S0021, S0025, S0030, S0031, S0033, S0040, S0041, S0043, S0050, S0051, S0053, S0080, S0081, S0083, S0090, S0091, S0093, S0100, S0101, S0103, S0110, S0111, S0113, S0120, S0121, S0123, S0130, S0131, S0133, S0140, S0141, S0143, S0150, S0151, S0153, S0180, S0181, S0183, S0190, S0191, S0193, S025X, S032X, S0510, S0511, S0512, S0520, S0521, S0522, S0530, S0531, S0532, S0540, S0541, S0542, S0560, S0561, S0562, S058X, S0590, S0591, S0592, S080X, S0811, S0812, S0881, S0889, S0912, S0920, S0921, S0922, S0930, S0939, S098X, S0990, S0991, S0993, S0140, S0300, S0301, S0302, S0303, S038X, S039X, S0340, S0341, S0342, S0343, S038X, S039X, S0401, S0402, S0403, S0404, S0410, S0411, S0412, S0420, S0421, S0422, S0430, S0431, S0432, S0440, S0441, S0442, S0450, S0451, S0452, S0460, S0461, S0462, S0470, S0471, S0472, S0481, S0489, S049X, S070X, S071X, S078X, S079X, S090X, S1500, S1501, S1502, S1509, S1510, S1511, S1512, S1519, S1520, S1521, S1522, S1529, S1530, S1531, S1532, S1539, S158X, S159X
Neck/Trunk Injuries	S2231, S2232, S2239, S2241, S2242, S2243, S2249, S3230, S3231, S3239, S3240, S3241, S3242, S3243, S3244, S3245, S3246, S3247, S3248, S3249, S3250, S3251, S3259, S3260, S3261, S3269, S3281, S3282, S3289, S329X, S1200, S1201, S1202, S1203, S1204, S1209, S1210, S1211, S1212, S1213, S1214, S1215, S1219, S1220, S1223, S1224, S1225, S1229, S1230, S1233, S1234, S1235, S1239, S1240, S1243, S1244, S1245, S1249, S1250, S1253, S1254, S1255, S1259, S1260, S1263, S1264, S1265, S1269, S128X, S129X, S1410, S1411, S1412, S1413, S1415, S2200, S2201, S2202, S2203, S2204, S2205, S2206, S2207, S2208, S2220, S2221, S2222, S2223, S2224, S225X, S229X, S2410, S2411, S2413, S2415, S3200, S3201, S3202, S3203, S3204, S3205, S3210, S3211, S3212, S3213, S3214, S3215, S3216, S3217, S3219, S322X, S3410, S3411, S3412, S3413, S343X, S1080, S1081, S1083, S1090, S1091, S1093, S1101, S1102, S1111, S1121, S1180, S1181, S1189, S1190, S1191, S1193, S162X, S2000, S2001, S2002, S2010, S2011, S2014, S2020, S2021, S2022, S2030, S2031, S2040, S2041, S2090, S2091, S2110, S2111, S2113, S2120, S2121, S2123, S2190, S2191, S2193, S2902, S300X, S301X, S3081, S3091, S3092, S3100, S3101, S3103, S3110, S3111, S3113, S3180, S3181, S3182, S383X, S3902, M9910, M9911, M9912, M9913, M9914, M9915, M9916, M9917, M9918, M9919, S1190, S130X, S1310, S1311, S1312, S1313, S1314, S1315, S1316, S1317, S1318, S1320, S1329, S2110, S2120, S230X, S2310, S2311, S2312, S2313, S2314, S2315, S2316, S2317, S2320, S2329, S3100, S330X, S3310, S3311, S3312, S3313, S3314, S332X, S3330, S3339, S4320, S4321, S4322, S038X, S039X, S134X, S138X, S139X, S161X, S233X, S2341, S2342, S238X, S239X, S2901, S334X, S335X,

© 2024 Wang GHM et al. *JAMA Network Open.*

Type of FRI	ICD-10-CM
	S336X, S338X, S339X, S3901, S2600, S2601, S2602, S2609, S2610, S2611, S2612, S2619, S2690, S2691, S2692, S2699, S270X, S271X, S272X, S2730, S2732, S2733, S2739, S2740, S2742, S2749, S2750, S2753, S2759, S2760, S2763, S2769, S2780, S2789, S279X, S3101, S3103, S3160, S3161, S3163, S3600, S3602, S3603, S3609, S3611, S3612, S3620, S3622, S3623, S3624, S3625, S3626, S3629, S3630, S3689, S3690, S3692, S3693, S3699, S3700, S3701, S3702, S3703, S3704, S3705, S3706, S3709, S3719, S3729, S3781, S140X, S1410, S1411, S1412, S1413, S1414, S1415, S142X, S143X, S145X, S170X, S178X, S179X, S240X, S2410, S2411, S2413, S2414, S2415, S242X, S243X, S244X, S248X, S249X, S280X, S3401, S3402, S3410, S3411, S3412, S3413, S3421, S3422, S343X, S344X, S345X, S346X, S348X, S349X, S358X
Upper Extremity Injuries	S4200, S4201, S4202, S4203, S4220, S4221, S4222, S4223, S4224, S4225, S4226, S4227, S4229, S4240, S4241, S4242, S4243, S4244, S4245, S4246, S4247, S4248, S4249, S4900, S4901, S4902, S4903, S4904, S4909, S4910, S4911, S4912, S4913, S4914, S4919, S4230, S4231, S4232, S4233, S4234, S4235, S4236, S4239, S4290, S4291, S4292, S5200, S5201, S5202, S5203, S5204, S5209, S5210, S5211, S5212, S5213, S5218, S5220, S5221, S5222, S5223, S5224, S5225, S5226, S5227, S5228, S5229, S5230, S5231, S5232, S5233, S5234, S5235, S5236, S5237, S5238, S5239, S5250, S5251, S5252, S5253, S5254, S5255, S5256, S5257, S5259, S5260, S5261, S5262, S5269, S5290, S5291, S5292, S5900, S5901, S5902, S5903, S5904, S5909, S5910, S5911, S5912, S5913, S5914, S5919, S5920, S5921, S5922, S5923, S5924, S5929, S6200, S6201, S6202, S6203, S6210, S6215, S6220, S6221, S6222, S6223, S6224, S6225, S6229, S6230, S6231, S6232, S6233, S6234, S6235, S6236, S6239, S6250, S6251, S6252, S6260, S6261, S6262, S6263, S6264, S6265, S6266, S6290, S6291, S6292, S4210, S4211, S4212, S4213, S4214, S4215, S4219, S6211, S6212, S6213, S6214, S6216, S6217, S6218, S4100, S4101, S4103, S4110, S4111, S4113, S4602, S4612, S4622, S4632, S4682, S4692, S5100, S5101, S5103, S5180, S5181, S5183, S5602, S5612, S5622, S5632, S5642, S5652, S5682, S5692, S6100, S6101, S6103, S6110, S6111, S6113, S6120, S6121, S6123, S6130, S6131, S6133, S6140, S6141, S6143, S6150, S6151, S6153, S6602, S6612, S6622, S6632, S6642, S6652, S6682, S6692, S4021, S4081, S4091, S4092, S5031, S5081, S5090, S5091, S6031, S6039, S6041, S6051, S6081, S6091, S6092, S6093, S6094, S4001, S4002, S5000, S5001, S5002, S5010, S5011, S5012, S6000, S6001, S6002, S6003, S6004, S6005, S6010, S6011, S6012, S6013, S6014, S6015, S6021, S6022, S4100, S4110, S4300, S4301, S4302, S4303, S4308, S4310, S4311, S4312, S4313, S4314, S4315, S4330, S4331, S4339, S5100, S5300, S5301, S5302, S5303, S5309, S5310, S5311, S5312, S5313, S5314, S5319, S6100, S6120, S6140, S6310, S6311, S6312, S6320, S6321, S6322, S6323, S6324, S6325, S6326, S6327, S6328, S6329, S4340, S4341, S4342, S4343, S4349, S4350, S4351, S4352, S4360, S4361, S4362, S4380, S4381, S4382, S4390, S4391, S4392, S4601, S4611, S4621, S4631, S4681, S4691, S6330, S6331, S6332, S6333, S6339, S6350, S6351, S6352, S6359, S6601, S6611, S6621, S6631, S6641, S6651, S6681, S6691, S6340, S6341, S6342, S6343, S6349, S6360, S6361, S6362, S6363, S6364, S6365, S6368, S6369, S638X, S6390, S6391, S6392, S4400, S4401, S4402, S4410, S4411, S4412, S4420, S4421, S4422, S4430, S4431, S4432, S4440, S4441, S4442, S4450, S4451, S4452, S448X, S4490, S4491, S4492, S4500, S4501, S4509, S4510, S4511, S4519, S4520, S4521, S4529, S4530, S4531, S4539, S4580, S4581, S4589, S4590, S4591,

© 2024 Wang GHM et al. *JAMA Network Open.*

Type of FRI	ICD-10-CM
	S4599, S471X, S472X, S479X, S5400, S5401, S5402, S5410, S5411, S5412, S5420, S5421, S5422, S5430, S5431, S5432, S548X, S5490, S5491, S5492, S5500, S5501, S5509, S5510, S5511, S5519, S5520, S5521, S5529, S5580, S5581, S5589, S5590, S5591, S5599, S5700, S5701, S5702, S5780, S5781, S5782, S6400, S6401, S6402, S6410, S6411, S6412, S6420, S6421, S6422, S6430, S6431, S6432, S6440, S6449, S648X, S6490, S6491, S6492, S6500, S6501, S6509, S6510, S6511, S6519, S6520, S6521, S6529, S6530, S6531, S6539, S6540, S6541, S6549, S6550, S6551, S6559, S6580, S6581, S6589, S6590, S6591, S6599, S6700, S6701, S6702, S6710, S6719, S6720, S6721, S6722, S6730, S6731, S6732, S6740, S6741, S6742, S6790, S6791, S6792
Lower Extremity Injuries	S7200, S7201, S7202, S7203, S7204, S7205, S7206, S7209, S7210, S7211, S7212, S7213, S7214, S7221, S7222, S7223, S7224, S7225, S7226, S7900, S7901, S7909, S7230, S7232, S7233, S7234, S7235, S7236, S7239, S7240, S7241, S7242, S7243, S7244, S7245, S7246, S7247, S7249, S728X, S7290, S7291, S7292, S7910, S7911, S7912, S7913, S7914, S7919, S8210, S8211, S8212, S8213, S8214, S8215, S8216, S8219, S8220, S8222, S8223, S8224, S8225, S8226, S8229, S8231, S8240, S8242, S8243, S8244, S8245, S8246, S8249, S8281, S8282, S8283, S8286, S8900, S8901, S8902, S8903, S8904, S8909, S8920, S8921, S8922, S8929, S8200, S8201, S8202, S8203, S8204, S8209, S8230, S8239, S8251, S8252, S8253, S8254, S8255, S8256, S8261, S8262, S8263, S8264, S8265, S8266, S8284, S8285, S8287, S8289, S8910, S8911, S8912, S8913, S8914, S8919, S8930, S8931, S8932, S8939, S8290, S8291, S8292, S9200, S9201, S9202, S9203, S9204, S9205, S9206, S9210, S9211, S9212, S9213, S9214, S9215, S9219, S9220, S9221, S9222, S9223, S9224, S9225, S9230, S9231, S9232, S9233, S9234, S9235, S9240, S9241, S9242, S9249, S9250, S9251, S9252, S9253, S9259, S9281, S9290, S9291, S9900, S9901, S9902, S9903, S9904, S9909, S9910, S9911, S9912, S9913, S9914, S9919, S9920, S9921, S9922, S9923, S9924, S9929, S7100, S7101, S7103, S7110, S7111, S7113, S7602, S7612, S7622, S7632, S7682, S7692, S8100, S8101, S8103, S8180, S8181, S8183, S8602, S8612, S8622, S8632, S8682, S8692, S9100, S9101, S9103, S9602, S9612, S9622, S9682, S9692, S9130, S9131, S9133, S9110, S9111, S9113, S9120, S9121, S9123, S7021, S7031, S7091, S7092, S8021, S8081, S8091, S8092, S9041, S9051, S9081, S9091, S9092, S9093, S7000, S7001, S7002, S7010, S7011, S7012, S8000, S8001, S8002, S8010, S8011, S8012, S9000, S9001, S9002, S9011, S9012, S9021, S9022, S9030, S9031, S9032, S7100, S7300, S7301, S7302, S7303, S7304, S7400, S7401, S7402, S7410, S7411, S7412, S7420, S7421, S7422, S748X, S7490, S7491, S7492, S7500, S7501, S7502, S7509, S7510, S7511, S7512, S7519, S7520, S7521, S7522, S7529, S7580, S7581, S7589, S7590, S7591, S7599, S7700, S7701, S7702, S7710, S7711, S7712, S7720, S7721, S7722, S8400, S8401, S8402, S8410, S8411, S8412, S8420, S8421, S8422, S8480, S8490, S8491, S8492, S8500, S8501, S8509, S8510, S8511, S8512, S8513, S8514, S8515, S8516, S8517, S8518, S8520, S8521, S8529, S8530, S8531, S8539, S8540, S8541, S8549, S8550, S8551, S8559, S8580, S8581, S8589, S8590, S8591, S8599, S8700, S8701, S8702, S8780, S8781, S8782, S9400, S9401, S9402, S9410, S9411, S9412, S9420, S9421, S9422, S9430, S9431, S9432, S948X, S9490, S9491, S9492, S9500, S9501, S9509, S9510, S9511, S9519, S9520, S9521, S9529, S9580, S9581, S9589, S9590, S9591, S9599, S9700, S9701, S9702, S9710, S9711, S9712, S9780, S9781, S9782
Fractures	S0190, S020X, S0210, S0211, S0219, S022X, S0230, S0231, S0232, S0240, S0241, S0242, S0260, S0261, S0262,

© 2024 Wang GHM et al. *JAMA Network Open.*

Type of FRI	ICD-10-CM
	S0263, S0264, S0265, S0266, S0267, S0269, S0280, S0281, S0282, S0291, S0292, S060X, S061X, S062X, S0630, S0631, S0632, S0633, S0634, S0635, S0636, S0637, S0638, S064X, S065X, S066X, S0681, S0682, S0689, S069X, S2231, S2232, S2239, S2241, S2242, S2243, S2249, S3230, S3231, S3239, S3240, S3241, S3242, S3243, S3244, S3245, S3246, S3247, S3248, S3249, S3250, S3251, S3259, S3260, S3261, S3269, S3281, S3282, S3289, S329X, S1200, S1201, S1202, S1203, S1204, S1209, S1210, S1211, S1212, S1213, S1214, S1215, S1219, S1220, S1223, S1224, S1225, S1229, S1230, S1233, S1234, S1235, S1239, S1240, S1243, S1244, S1245, S1249, S1250, S1253, S1254, S1255, S1259, S1260, S1263, S1264, S1265, S1269, S128X, S129X, S1410, S1411, S1412, S1413, S1415, S2200, S2201, S2202, S2203, S2204, S2205, S2206, S2207, S2208, S2220, S2221, S2222, S2223, S2224, S225X, S229X, S2410, S2411, S2413, S2415, S3200, S3201, S3202, S3203, S3204, S3205, S3210, S3211, S3212, S3213, S3214, S3215, S3216, S3217, S3219, S322X, S3410, S3411, S3412, S3413, S343X, S4200, S4201, S4202, S4203, S4220, S4221, S4222, S4223, S4224, S4225, S4226, S4227, S4229, S4240, S4241, S4242, S4243, S4244, S4245, S4246, S4247, S4248, S4249, S4900, S4901, S4902, S4903, S4904, S4909, S4910, S4911, S4912, S4913, S4914, S4919, S4230, S4231, S4232, S4233, S4234, S4235, S4236, S4239, S4290, S4291, S4292, S5200, S5201, S5202, S5203, S5204, S5209, S5210, S5211, S5212, S5213, S5218, S5220, S5221, S5222, S5223, S5224, S5225, S5226, S5227, S5228, S5229, S5230, S5231, S5232, S5233, S5234, S5235, S5236, S5237, S5238, S5239, S5250, S5251, S5252, S5253, S5254, S5255, S5256, S5257, S5259, S5260, S5261, S5262, S5269, S5290, S5291, S5292, S5900, S5901, S5902, S5903, S5904, S5909, S5910, S5911, S5912, S5913, S5914, S5919, S5920, S5921, S5922, S5923, S5924, S5929, S6200, S6201, S6202, S6203, S6210, S6215, S6220, S6221, S6222, S6223, S6224, S6225, S6229, S6230, S6231, S6232, S6233, S6234, S6235, S6236, S6239, S6250, S6251, S6252, S6260, S6261, S6262, S6263, S6264, S6265, S6266, S6290, S6291, S6292, S4210, S4211, S4212, S4213, S4214, S4215, S4219, S6211, S6212, S6213, S6214, S6216, S6217, S6218, S7200, S7201, S7202, S7203, S7204, S7205, S7206, S7209, S7210, S7211, S7212, S7213, S7214, S7221, S7222, S7223, S7224, S7225, S7226, S7900, S7901, S7909, S7230, S7232, S7233, S7234, S7235, S7236, S7239, S7240, S7241, S7242, S7243, S7244, S7245, S7246, S7247, S7249, S728X, S7290, S7291, S7292, S7910, S7911, S7912, S7913, S7914, S7919, S8210, S8211, S8212, S8213, S8214, S8215, S8216, S8219, S8220, S8222, S8223, S8224, S8225, S8226, S8229, S8231, S8240, S8242, S8243, S8244, S8245, S8246, S8249, S8281, S8282, S8283, S8286, S8900, S8901, S8902, S8903, S8904, S8909, S8920, S8921, S8922, S8929, S8200, S8201, S8202, S8203, S8204, S8209, S8230, S8239, S8251, S8252, S8253, S8254, S8255, S8256, S8261, S8262, S8263, S8264, S8265, S8266, S8284, S8285, S8287, S8289, S8910, S8911, S8912, S8913, S8914, S8919, S8930, S8931, S8932, S8939, S8290, S8291, S8292, S9200, S9201, S9202, S9203, S9204, S9205, S9206, S9210, S9211, S9212, S9213, S9214, S9215, S9219, S9220, S9221, S9222, S9223, S9224, S9225, S9230, S9231, S9232, S9233, S9234, S9235, S9240, S9241, S9242, S9249, S9250, S9251, S9252, S9253, S9259, S9281, S9290, S9291, S9900, S9901, S9902, S9903, S9904, S9909, S9910, S9911, S9912, S9913, S9914, S9919, S9920, S9921, S9922, S9923, S9924, S9929
**Exclusion**	

© 2024 Wang GHM et al. *JAMA Network Open.*

Type of FRI	ICD-10-CM
Bone Cancer	C400, C401, C410, C411, C412, C413, C414, C418, C402, C403, C409, C408, C419, C7951, C7952
Motor vehicle accidents	V00, V01, V02, V03, V04, V05, V06, V07, V08, V09, V10, V11, V12, V13, V14, V15, V16, V17, V18, V19, V20, V21, V22, V23, V24, V25, V26, V27, V28, V29, V30, V31, V32, V33, V34, V35, V36, V37, V38, V39, V40, V41, V42, V43, V44, V45, V46, V47, V48, V49, V50, V51, V52, V53, V54, V55, V56, V57, V58, V59, V60, V61, V62, V63, V64, V65, V66, V67, V68, V69, V70, V71, V72, V73, V74, V75, V76, V77, V78, V79, V80, V81, V82, V83, V84, V85, V86, V87, V88, V89, V90, V91, V92, V93, V94, V95, V96, V97, V98, V99
Contact with other objects, gunshots, drowning, electrocutions	W20, W21, W22, W23, W24, W25, W26, W27, W28, W29, W30, W31, W32, W33, W34, W35, W36, W37, W38, W39, W40, W41, W42, W43, W44, W45, W46, W47, W48, W49, W50, W51, W52, W53, W54, W55, W56, W57, W58, W59, W60, W61, W62, W63, W64, W65, W66, W67, W68, W69, W70, W71, W72, W73, W74, W75, W76, W77, W78, W79, W80, W81, W82, W83, W84, W85, W86, W87, W88, W89, W90, W91, W92, W93, W94, W95, W96, W97, W98, W99
Burns and exposure	X00, X01, X02, X03, X04, X05, X06, X07, X08, X09, X10, X11, X12, X13, X14, X15, X16, X17, X18, X19, X20, X21, X22, X23, X24, X25, X26, X27, X28, X29, X30, X31, X32, X33, X34, X35, X36, X37, X38, X39, X40, X41, X42, X43, X44, X45, X46, X47, X48, X49, X50, X51, X52, X53, X54, X55, X56, X57, X58, X59, X60, X61, X62, X63, X64, X65, X66, X67, X68, X69, X70, X71, X72, X73, X74, X75, X76, X77, X78, X79, X80, X81, X82, X83, X84, X85, X86, X87, X88, X89, X90, X91, X92, X93, X94, X95, X96, X97, X98, X99
Self-harm	Y00, Y01, Y02, Y03, Y04, Y05, Y06, Y07, Y08, Y09, Y10, Y11, Y12, Y13, Y14, Y15, Y16, Y17, Y18, Y19, Y20, Y21, Y22, Y23, Y24, Y25, Y26, Y27, Y28, Y29, Y30, Y31, Y32, Y33, Y34, Y35, Y36, Y37, Y38, Y39, Y40, Y41, Y42, Y43, Y44, Y45, Y46, Y47, Y48, Y49, Y50, Y51, Y52, Y53, Y54, Y55, Y56, Y57, Y58, Y59, Y60, Y61, Y62, Y63, Y64, Y65, Y66, Y67, Y68, Y69, Y70, Y71, Y72, Y73, Y74, Y75, Y76, Y77, Y78, Y79, Y80, Y81, Y82, Y83, Y84, Y85, Y86, Y87, Y88, Y89, Y90, Y91, Y92, Y93, Y94, Y95, Y96, Y97, Y98, Y99

© 2024 Wang GHM et al. *JAMA Network Open.*

eTable 3. International Classification of Diseases Clinical Modification Codes for Comorbidities

Clinical characteristics	ICD-10-CM
Alcohol abuse/dependence	F101, F102
Anxiety disorder	F430, R457, F41, F40, F42, F488, F489, F99, F938, F064, F449, F431
Bipolar disorder	F30, F31
Chronic pain	
Abdominal pain/hernia	K37, K3580, K3589, K429, K449, K581, K582, K588, K589, K8590, K8591, K8592, K279, K2900, K4090, K4020, K439, K432, K469, N12, R109, R1011, R1012, R1031, R1032, R1033, R1013, R1084, R1010, R102, R1030
Back pain	M4000, M40209, M40299, M404, M405, M4100, M4120, M4130, M4180, M419, M4300, M4310, M4327, M4328, M4389, M459, M4600, M461, M4640, M4645, M4647, M465, M468, M469, M4714, M4715, M4716, M4800, M4804, M4806, M4808, M481, M482, M483, M489, M498, M5104, M5105, M5106, M512, M513, M5136, M5137, M5146, M5147, M518, M519, M5327, M5328, M533, M539, M5408, M5414, M5415, M5416, M5417, M5430, M545, M546, M5489, M549, M961, M962, M963, M964, M965, M9902, M9903, M9904, M9983, M9984, Q760, Q761, Q762, Q76419, Q7649, S129A, S22009A, S23101A, S233A, S238A, S239A, S32009A, S3210A, S322A, S33101A, S332A, S335A, S336A, S338A, S339A
Catastrophizing	F454
Chest pain	I208, I209, R079, R072, R071, R0781, R0782, R0789
Internal orthopedic device implant and graft	M979A, T84019A, T84029A, T84039A, T84059A, T84069A, T84099A, T84119A, T84129A, T84199A, T84498A
Fibromyalgia	M609, M791, M797
Headache/migraine	G43019, G43109, G43119, G43711, G43809, G43819, G43909, G43919, G43A, G43B, G43C, G43D, G441, R51, G44209
Injury	M4850A, M8008A, M8448A, M8468A, S0001A , S0031A, S00419A, S00511A , S00512A , S0091A , S0093A, S0100A, S01501A, S022A, S0600A, S098A, S0990A , S1011A , S1091A, S1093A, S20219A , S22009A, S2220A , S2239A, S298A, S300A, S300A , S301A, S31000A, S329A, S3981A, S3982A , S39840A, S39848A, S40019A, S40019A , S42009A, S42209A , S42293A, S42296A, S42309A, S42409A, S43109A, S4980A , S4990A, S5000A, S5010A, S50319A, S50819A , S51009A , S51809A , S52023A, S52026A , S52123A , S52126A, S52539A, S52549A, S5290A, S6000A , S60019A , S6010A, S60219A, S60229A, S60519A, S60819A, S61109A, S61209A, S61409A , S62009A, S62109A , S62309A, S62509A, S62509B , S62523A, S62526A , S62609A , S62609B, S62639A , S62669A, S66529A, S6700A, S6710A, S68119A, S68129A, S68619A, S68629A, S7000A, S7010A, S70219A , S70319A, S72009A , S8000A , S8010A , S80819A, S81009A, S81809A , S82009A, S82109A, S82201A, S82401A, S8253A, S8256A , S8263A, S8266A, S82843A, S82846A , S82853A , S82856A, S82899A , S8980A, S8990A , S9000A, S90119A, S90129A, S90229A, S9030A, S90519A, S91009A, S91109A , S91309A, S92009A, S92309A,

© 2024 Wang GHM et al. *JAMA Network Open.*

Clinical characteristics	ICD-10-CM
	S92403A, S92406A , S92503A, S92506A , S92819A , S92909A, S99009A, S99019A , S99029A , S99039A, S99049A, S99099A , S99109A, S99119A , S99129A, S99139A, S99149A , S99199A , S99819A , S99919A, T07A , T148A, T148A , T1490A, W01110A, W01198A, W1830A , W1849A, W19A, W540A , W5501A
Neck pain	M436, M4712, M47812, M4802, M5000, M5020, M5030, M5080, M5090, M5144, M5145, M530, M531, M5382, M5402, M5412, M5413, M542, M6788, M961, S1190A, S13101A, S13111A, S13121A, S13131A, S13141A, S13151A, S13161A, S13171A, S13181A, S134A, S138A
Osteoarthritis	D481, M0000, M00019, M00029, M0010, M00119, M00129, M0020, M00219, M00229, M0080, M00819, M00829, M009, M020, M118, M125, M1480, M150, M151, M152, M153, M158, M159, M1610, M167, M169, M1710, M175, M179, M189, M190, M192, M1990, M1991, M1993, M200, M201, M202, M203, M204, M205, M206, M21, M214, M40, M41, M42 , M430, M431, M438, M462, M463, M47814, M47817, M47819, M48, M651, M653, M654, M658, M6580, M659, M80, M81, M84, M85, M861, M862, M866, M869, M87, M88, M89, M8938, M894, M896, M8988, M905, M906, M908, M91, M92, M93, M94, M95, M962, M963, M964, M99, M998, M999, S02, S12, S22, S32, S42, S49, S52, S62, S68, S72, S82, S92
Menstrual/genital reproductive pain	N946, N9489, R102, N921, N950, N951
Rheumatoid arthritis	M05, M06, M080, M082, M083, M088, M089, M120
Kidney/gall bladder stones	N200, N201, N209, N210, K8018, K8020, K819
Temporomandibular disorder	M26601, M26602, M26603, M26609, M26611, M26612, M26613, M26619, M26621, M26622, M26623, M26629, M26631, M26632, M26633, M26639, M2669
Others	M6747, M7030, D481, H5713, H60509, H60519 , H60529 , H60539, H60549 , H60559 , H60599, H6060, H6081 , H6090, H6690, H6980, H9209, J342 , K044, K047, K089, K603, K604, K605, K6289, L600 , L723 , M1230, M1240 , M2010, M2161 , M2162 , M2420 , M2570, M25729 , M353, M357, M5410, M60009 , M6010 , M6020, M609, M6100, M6110 , M6140 , M6159 , M619, M6200 , M6210, M623, M6240, M6250 , M62838 , M6284, M6289, M629 , M6500, M6520, M6530 , M654 , M6580, M65849 , M65879, M659 , M6610, M6618 , M66239, M66249 , M66259, M66269, M66339 , M66349, M66369 , M66829, M66879, M6688 , M669 , M6700, M6740, M6741, M6742 , M6743 , M6744, M6745 , M6746 , M6750, M6780, M6788 , M6790 , M70039 , M701, M702, M703, M7040, M705, M706, M707, M7098 , M7100 , M7120 , M7130, M7140 , M7150, M7180, M719 , M720 , M721 , M722 , M724, M726 , M729, M750, M7510, M75120, M752, M753, M754, M755, M758, M7610 , M7620, M7640 , M7650, M7660 , M76829 , M76899, M7700, M7710 , M7720 , M7730, M7740 , M7750, M778 , M779 , M790, M791 , M792, M793 , M794, M795 , M79609, M797, M7981, M7989, M799, N451, N452, N453 , N644 , N6451, N6452, N6453, N6459, R071, R0781, R252, R29898 , S025A, S025B, S039A, S2341A , S29019A, S39011A , T1490A

© 2024 Wang GHM et al. *JAMA Network Open.*

Clinical characteristics	ICD-10-CM
Congestive heart failure	I0981, I501, I5020, I5021, I5022, I5023, I5030, I5031, I5032, I5033, I5040, I5041, I5042, I5043, I50810, I50811, I50812, I50813, I50814, I5082, I5083, I5084, I5089, I509, I5181, I97130, I97131, O29121, O29122, O29123, O29129, R570, Z95811, Z95812
Dementia	F0150, F0151, F0152, F0153, F0154, F01A0, F01A11, F01A18, F01A2, F01A3, F01A4, F01B0, F01B11, F01B18, F01B2, F01B3, F01B4, F01C0, F01C11, F01C18, F01C2, F01C3, F01C4, F0280, F0281, F0282, F0283, F0284, F02A0, F02A11, F02A18, F02A2, F02A3, F02A4, F02B0, F02B11, F02B18, F02B2, F02B3, F02B4, F02C0, F02C11, F02C18, F02C2, F02C3, F02C4, F0390, F0391, F0392, F0393, F0394, F03A0, F03A11, F03A18, F03A2, F03A3, F03A4, F03B0, F03B11, F03B18, F03B2, F03B3, F03B4, F03C0, F03C11, F03C18, F03C2, F03C3, F03C4, F0670, F0671, G300, G301, G308, G309, G3101, G3109, G311, G312, G3181, G3182, G3183, G3185, G3189, G319
Diabetes	E0800, E0801, E0810, E0811, E089, E0900, E0901, E0910, E0911, E099, E1010, E1011, E109, E1100, E1101, E1110, E1111, E119, E1300, E1301, E1310, E1311, E139, O24011, O24012, O24013, O24019, O2402, O2403, O24111, O24112, O24113, O24119, O2412, O2413, O24311, O24312, O24313, O24319, O2432, O2433, O24410, O24414, O24415, O24419, O24420, O24424, O24425, O24429, O24430, O24434, O24435, O24439, O24811, O24812, O24813, O24819, O2482, O2483, O24911, O24912, O24913, O24919, O2492, O2493, E0821, E0822, E0829, E08311, E08319, E08321, E08329, E08331, E08339, E08341, E08349, E08351, E083521, E083522, E083523, E083529, E083531, E083532, E083533, E083539, E083541, E083542, E083543, E083549, E083551, E083552, E083553, E083559, E08359, E0836, E0837X1, E0837X2, E0837X3, E0837X9, E0839, E0840, E0841, E0842, E0843, E0844, E0849, E0851, E0852, E0859, E08610, E08618, E08620, E08621, E08622, E08628, E08630, E08638, E08641, E08649, E0865, E0869, E088, E0921, E0922, E0929, E09311, E09319, E09321, E09329, E09331, E09339, E09341, E09349, E09351, E093521, E093522, E093523, E093529, E093531, E093532, E093533, E093539, E093541, E093542, E093543, E093549, E093551, E093552, E093553, E093559, E09359, E0936, E0937X1, E0937X2, E0937X3, E0937X9, E0939, E0940, E0941, E0942, E0943, E0944, E0949, E0951, E0952, E0959, E09610, E09618, E09620, E09621, E09622, E09628, E09630, E09638, E09641, E09649, E0965, E0969, E098, E1021, E1022, E1029, E10311, E10319, E10321, E10329, E10331, E10339, E10341, E10349, E103491, E10351, E103521, E103522, E103523, E103529, E103531, E103532, E103533, E103539, E103541, E103542, E103543, E103549, E103551, E103552, E103553, E103559, E10359, E1036, E1037X1, E1037X2, E1037X3, E1037X9, E1039, E1040, E1041, E1042, E1043, E1044, E1049, E1051, E1052, E1059, E10610, E10618, E10620, E10621, E10622, E10628, E10630, E10638, E10641, E10649, E1065, E1069, E108, E1121, E1122, E1129, E11311, E11319, E11321, E11329, E11331, E11339, E11341, E11349, E11351, E113521, E113522, E113523, E113529, E113531, E113532, E113533, E113539, E113541, E113542, E113543, E113549, E113551, E113552, E113553, E113559, E11359, E1136, E1137X1, E1137X2, E1137X3, E1137X9, E1139, E1140, E1141, E1142, E1143, E1144, E1149, E1151,

© 2024 Wang GHM et al. *JAMA Network Open.*

Clinical characteristics	ICD-10-CM
	E1152, E1159, E11610, E11618, E11620, E11621, E11622, E11628, E11630, E11638, E11641, E11649, E1165, E1169, E118, E1321, E1322, E1329, E13311, E13319, E13321, E13329, E13331, E13339, E13341, E13349, E13351, E133521, E133522, E133523, E133529, E133531, E133532, E133533, E133539, E133541, E133542, E133543, E133549, E133551, E133552, E133553, E133559, E13359, E1336, E1337X1, E1337X2, E1337X3, E1337X9, E1339, E1340, E1341, E1342, E1343, E1344, E1349, E1351, E1352, E1359, E13610, E13618, E13620, E13621, E13622, E13628, E13630, E13638, E13641, E13649, E1365, E1369, E138
Drug abuse/dependence	F1110, F1111, F11120, F11121, F11122, F11129, F1113, F1114, F11150, F11151, F11159, F11181, F11182, F11188, F1119, F1120, F1121, F11220, F11221, F11222, F11229, F1123, F1124, F11250, F11251, F11259, F11281, F11282, F11288, F1129, F1210, F1211, F12120, F12121, F12122, F12129, F1213, F12150, F12151, F12159, F12180, F12188, F1219, F1220, F1221, F12220, F12221, F12222, F12229, F1223, F12250, F12251, F12259, F12280, F12288, F1229, F1310, F1311, F13120, F13121, F13129, F13130, F13131, F13132, F13139, F1314, F13150, F13151, F13159, F13180, F13181, F13182, F13188, F1319, F1320, F1321, F13220, F13221, F13229, F13230, F13231, F13232, F13239, F1324, F13250, F13251, F13259, F1326, F1327, F13280, F13281, F13282, F13288, F1329, F1410, F1411, F14120, F14121, F14122, F14129, F1413, F1414, F14150, F14151, F14159, F14180, F14181, F14182, F14188, F1419, F1420, F1421, F14220, F14221, F14222, F14229, F1423, F1424, F14250, F14251, F14259, F14280, F14281, F14282, F14288, F1429, F1510, F1511, F15120, F15121, F15122, F15129, F1513, F1514, F15150, F15151, F15159, F15180, F15181, F15182, F15188, F1519, F1520, F1521, F15220, F15221, F15222, F15229, F1523, F1524, F15250, F15251, F15259, F15280, F15281, F15282, F15288, F1529, F1610, F1611, F16120, F16121, F16122, F16129, F1614, F16150, F16151, F16159, F16180, F16183, F16188, F1619, F1620, F1621, F16220, F16221, F16229, F1624, F16250, F16251, F16259, F16280, F16283, F16288, F1629, F1810, F1811, F18120, F18121, F18129, F1814, F18150, F18151, F18159, F1817, F18180, F18188, F1819, F1820, F1821, F18220, F18221, F18229, F1824, F18250, F18251, F18259, F1827, F18280, F18288, F1829, F1910, F1911, F19120, F19121, F19122, F19129, F19130, F19131, F19132, F19139, F1914, F19150, F19151, F19159, F1916, F1917, F19180, F19181, F19182, F19188, F1919, F1920, F1921, F19220, F19221, F19222, F19229, F19230, F19231, F19232, F19239, F1924, F19250, F19251, F19259, F1926, F1927, F19280, F19281, F19282, F19288, F1929, O99320, O99321, O99322, O99323, O99324, O99325
Epilepsy	G40
Hyperlipidemia	E78
Hypertension	H35031, H35032, H35033, H35039, I110, I119, I120, I129, I130, I1310, I1311, I132, I150, I151, I152, I158, I159, I161, I674, O10111, O10112, O10113, O10119, O1012, O1013, O10211, O10212, O10213, O10219, O1022, O1023, O10311, O10312, O10313, O10319, O1032, O1033, O10411, O10412, O10413, O10419, O1042, O1043, O10911, O10912, O10913, O10919, O1092, O1093, O111, O112, O113, O114, O115,

© 2024 Wang GHM et al. *JAMA Network Open.*

Clinical characteristics	ICD-10-CM
	O119, O161, O162, O163, O164, O165, O169, I10, I160, I169, O10011, O10012, O10013, O10019, O1002, O1003
Hypotension	I95
Ischemic heart disease	I200, I201, I208, I209, I2109, I2111, I2119, I2129, I213, I214, I219, I21A1, I21A9, I240, I241, I248, I2510, I252, I2542, I255, I25810, I25811, I25812, I2582, I2583, I2584 , I2589, I259
Osteoporosis	M80, M81
Parkinson's disease/parkinsonism	G20, G21
Schizophrenia	F200, F201, F202, F205, F2081, F2089, F209, F259
Stroke or cerebrovascular accident	I60, I61, I62, I63, I64, G463, G464
Syncope	G9001, R054, R55, T671
Urinary incontinence	R32, R3981, N393, N394
Vertigo/dizziness	A881, H81, H82, R42, T7523
Vision disorders	H00-H59

© 2024 Wang GHM et al. *JAMA Network Open.*

eTable 4. FRI rates, RMST, and HRs for Sensitivity analysis (FRI=inclusive algorithm, grace period=90 days, follow-up=365 days)

	Event rate per 1,000 person-year (95% CI)	RMST (95% CI)	HR (95% CI)
No treatment	218 (212, 224)	322 (321, 324)	REF
Psychotherapy	209 (192, 223)	324 (321, 327)	0.94 (0.87, 1.02)
Sertraline	172 (154, 182)	332 (330, 335)	0.76 (0.69, 0.83)
Escitalopram	175 (161, 182)	331 (329, 335)	0.78 (0.72, 0.82)
Citalopram	171 (156, 180)	332 (329, 334)	0.77 (0.70, 0.81)
Mirtazapine	175 (163, 187)	331 (328, 333)	0.78 (0.73, 0.85)
Duloxetine	172 (159, 182)	331 (329, 334)	0.78 (0.72, 0.81)
Trazodone	169 (152, 181)	332 (329, 335)	0.76 (0.68, 0.81)
Fluoxetine	167 (156, 180)	332 (330, 334)	0.75 (0.70, 0.80)
Bupropion	166 (155, 174)	332 (331, 335)	0.74 (0.70, 0.78)
Paroxetine	167 (152, 173)	332 (331, 335)	0.74 (0.68, 0.77)
Venlafaxine	167 (152, 176)	332 (330, 335)	0.75 (0.68, 0.79)

Abbreviations: FRI: fall and related injuries, RMST: restricted mean survival time, HR: hazards ratio, CI: confidence interval, REF: reference

© 2024 Wang GHM et al. *JAMA Network Open.*

eTable 5. FRI rates, RMST, and HRs for sensitivity analysis (FRI=acute care algorithm, grace period=90 days, follow-up=183 days)

	Event rate per 1,000 person-year (95% CI)	RMST (95% CI)	HR (95% CI)
No treatment	46 (44, 49)	179 (177, 179)	REF
Psychotherapy	41 (33, 46)	179 (178, 180)	0.89 (0.69, 1.04)
Sertraline	38 (31, 44)	180 (178, 180)	0.82 (0.65, 1.02)
Escitalopram	38 (33, 46)	179 (178, 180)	0.83 (0.70, 0.99)
Citalopram	38 (31, 43)	180 (178, 180)	0.83 (0.64, 0.92)
Mirtazapine	37 (32, 44)	180 (178, 180)	0.79 (0.66, 0.91)
Duloxetine	36 (31, 40)	180 (178, 180)	0.79 (0.64, 0.89)
Trazodone	36 (29, 41)	180 (178, 180)	0.77 (0.61, 0.93)
Fluoxetine	35 (29, 40)	180 (178, 180)	0.77 (0.60, 0.87)
Bupropion	34 (27, 39)	180 (178, 181)	0.74 (0.57, 0.85)
Paroxetine	35 (28, 39)	180 (178, 181)	0.76 (0.57, 0.87)
Venlafaxine	34 (28, 40)	180 (178, 181)	0.75 (0.58, 0.89)

Abbreviations: FRI: fall and related injuries, RMST: restricted mean survival time, HR: hazards ratio, CI: confidence interval, REF: reference

© 2024 Wang GHM et al. *JAMA Network Open.*

eTable 6. FRI rates, RMST, and HRs for sensitivity analysis (FRI=acute care algorithm, grace period=30 days, follow-up=365 days)

	Event rate per 1,000 person-year (95% CI)	RMST (95% CI)	HR (95% CI)
No treatment	88 (85, 91)	348 (346, 349)	REF
Psychotherapy	82 (62, 102)	348 (345, 353)	0.92 (0.67, 1.20)
Sertraline	60 (47, 73)	353 (350, 356)	0.67 (0.52, 0.84)
Escitalopram	62 (50, 73)	353 (349, 355)	0.68 (0.56, 0.84)
Citalopram	63 (45, 76)	353 (349, 356)	0.71 (0.49, 0.88)
Mirtazapine	59 (51, 70)	353 (351, 355)	0.65 (0.56, 0.79)
Duloxetine	63 (52, 79)	352 (349, 355)	0.72 (0.56, 0.93)
Trazodone	60 (47, 70)	353 (350, 356)	0.66 (0.52, 0.79)
Fluoxetine	61 (49, 72)	353 (350, 355)	0.66 (0.53, 0.83)
Bupropion	54 (43, 65)	354 (351, 357)	0.61 (0.47, 0.73)
Paroxetine	59 (45, 73)	353 (351, 356)	0.67 (0.50, 0.82)
Venlafaxine	59 (49, 74)	353 (351, 356)	0.65 (0.54, 0.83)

Abbreviations: FRI: fall and related injuries, RMST: restricted mean survival time, HR: hazards ratio, CI: confidence interval, REF: reference

© 2024 Wang GHM et al. *JAMA Network Open.*

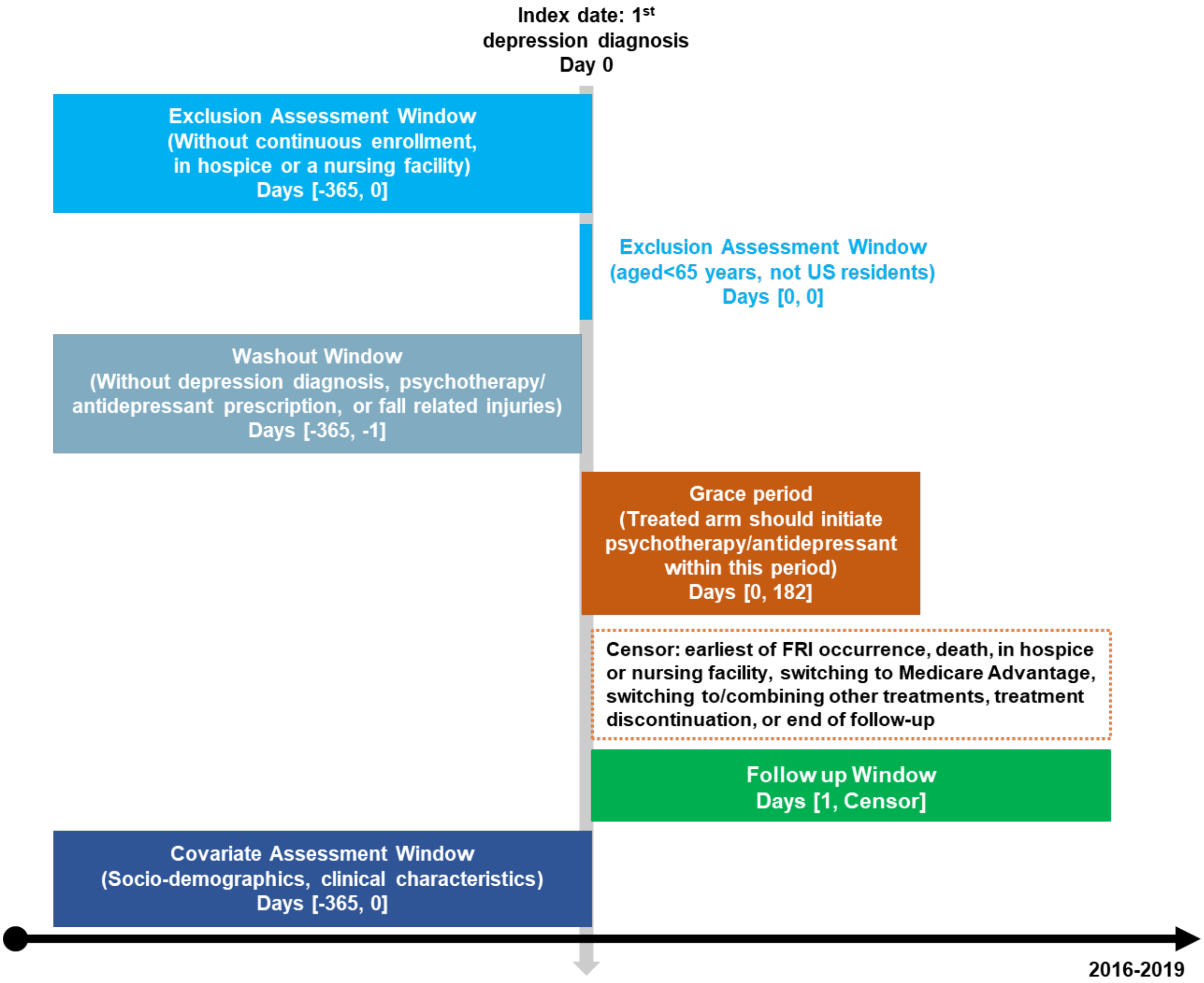

eFigure 1. Study Design Schematic Diagram

© 2024 Wang GHM et al. *JAMA Network Open.*

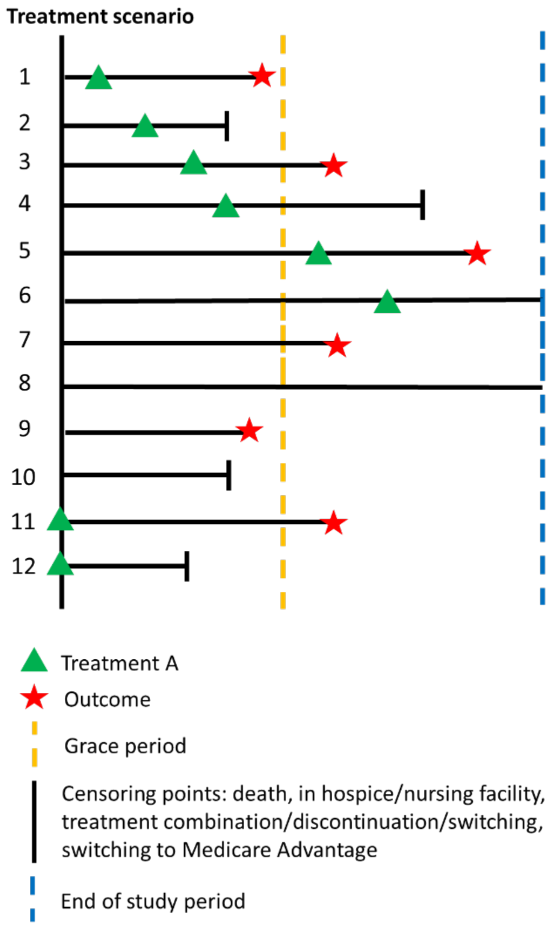

Both the “censoring-weighting” and the “outcome analysis” models were built using the multivariable Cox proportional hazards regression
T: Time to event
$\boldsymbol{\delta_D}$: Event in the “censoring-weighting” model (i.e., treatment deviation)
$\boldsymbol{\delta_O}$: Event in the “outcome analysis” model (i.e., falls and related injuries [FRI])
$\mathbf{T_A}$: Time to treatment A initiation
$\mathbf{T_G}$: Time to the end of the grace period
$\mathbf{T_O}$: Time to the outcome (i.e., FRI)
$\mathbf{T_C}$: Time to the censoring point
IPCW: inverse-probability-of-censoring weighting

	Step 1. Cloning	Step 2. Censoring-Weighting		Step 3. Outcome Analysis	
Scenario		δ_D	T	δ_O	T
1	Treatment A	0	T_A	1	T_O
	Other treatments (n=10)	1	T_A	0	T_A
	Untreated	1	T_A	0	T_A
2	Treatment A	0	T_A	0	T_C
	Other treatments (n=10)	1	T_A	0	T_A
	Untreated	1	T_A	0	T_A
3	Treatment A	0	T_A	1	T_O
	Other treatments (n=10)	1	T_A	0	T_A
	Untreated	1	T_A	0	T_A
4	Treatment A	0	T_A	0	T_C
	Other treatments (n=10)	1	T_A	0	T_A
	Untreated	1	T_A	0	T_A
5	Treatment A	1	T_G	0	T_G
	Other treatments (n=10)	1	T_G	0	T_G
	Untreated	0	T_G	1	T_O
6	Treatment A	1	T_G	0	T_G
	Other treatments (n=10)	1	T_G	0	T_G
	Untreated	0	T_G	0	T_C
7	Treatment A	1	T_G	0	T_G
	Other treatments (n=10)	1	T_G	0	T_G
	Untreated	0	T_G	1	T_O
8	Treatment A	1	T_G	0	T_G
	Other treatments (n=10)	1	T_G	0	T_G
	Untreated	0	T_G	0	T_C
9	Treatment A	0	T_O	1	T_O
	Other treatments (n=10)	0	T_O	1	T_O
	Untreated	0	T_O	1	T_O
10	Treatment A	0	T_C	0	T_C
	Other treatments (n=10)	0	T_C	0	T_C
	Untreated	0	T_C	0	T_C
11	Treatment A *No cloning	IPCW=1		1	T_O
12	Treatment A *No cloning	IPCW=1		0	T_G

eFigure 2. Treatment scenarios assignment, censoring weighting and outcome analysis in the targeted trial emulation approach.

© 2024 Wang GHM et al. *JAMA Network Open.*

REFERENCES

1. Hernán MA. How to estimate the effect of treatment duration on survival outcomes using observational data. *Bmj*. 2018;360

2. Hernán MA, Robins JM. Using big data to emulate a target trial when a randomized trial is not available. *American journal of epidemiology*. 2016;183(8):758-764.

3. Robinson RL, Long SR, Chang S, et al. Higher costs and therapeutic factors associated with adherence to NCQA HEDIS antidepressant medication management measures: analysis of administrative claims. *Journal of Managed Care Pharmacy*. 2006;12(1):43-54.

4. Kupfer DJ. Long-term treatment of depression. *The Journal of clinical psychiatry*. 1991;52:28-34.

5. Willems S, Schat A, van Noorden M, Fiocco M. Correcting for dependent censoring in routine outcome monitoring data by applying the inverse probability censoring weighted estimator. *Statistical methods in medical research*. 2018;27(2):323-335.

6. SCHOENFELD D. Partial residuals for the proportional hazards regression model. *Biometrika*. 1982;69(1):239-241. doi:10.1093/biomet/69.1.239

7. Calkins KL, Canan CE, Moore RD, Lesko CR, Lau B. An application of restricted mean survival time in a competing risks setting: comparing time to ART initiation by injection drug use. *BMC medical research methodology*. 2018;18:1-10.

8. Han K, Jung I. Restricted mean survival time for survival analysis: a quick guide for clinical researchers. *Korean Journal of Radiology*. 2022;23(5):495.

9. Zucker DM. Restricted mean life with covariates: modification and extension of a useful survival analysis method. *Journal of the American Statistical Association*. 1998;93(442):702-709.

10. Altman DG, Bland JM. Interaction revisited: the difference between two estimates. *Bmj*. 2003;326(7382):219.

© 2024 Wang GHM et al. *JAMA Network Open.*
