# Supplementary material for: Injurious Fall Risk Differences Among Older Adults With First-Line Depression Treatments
Source: JAMA Netw Open. 2024 Aug 26;7(8):e2435535. doi: 10.1001/jamanetworkopen.2024.35535 (PMC12068830; doi:10.1001/jamanetworkopen.2024.35535)
Supplement: Supplement 2. — Data Sharing Statement [file jamanetwopen-e2435535-s002.pdf]

Data Sharing Statement

Wang. Injurious Fall Risk Differences Among Older Adults With First-Line Depression Treatments. *JAMA Netw Open*. Published August 26, 2024. doi:10.1001/jamanetworkopen.2024.35535

Data
Data available: No

Additional Information
Explanation for why data not available: Medicare data are available from the Research Data AssistanceCenter (ResDAC) under data use agreement provisions. Per the data use agreement,the relevant limited data sets cannot be made publicly available.
